# Supplementary material for: New Steroidal Saponins Isolated from the Rhizomes of Paris mairei
Source: Molecules. 2021 Oct 21;26(21):6366. doi: 10.3390/molecules26216366 (PMC8588014; doi:10.3390/molecules26216366)

Supporting Information

New steroidal saponins from the rhizomes of *Paris mairei*

Yang Liu [1], Pengcheng Qiu [2], Minchang Wang [3,4], Yunyang Lu [2], Hao He [5],

Haifeng Tang [2,*] and Bangle Zhang [1,*]

[1] Department of Pharmaceutics, School of Pharmacy, Air Force Medical University, Xi'an, China; so870823@163.com (Y.L.)

[2] Department of Chinese Materia Medica and Natural Medicines, School of Pharmacy, Air Force Medical University, Xi'an, China; qpc023@126.com (P.Q.); luyunyanggq@163.com (Y.L.)

[3] Xi'an Modern Chemistry Research Institute,Xi'an 710065,China; wmc204@163.com (M.W.)

[4] State Key Laboratory of Fluorine&Nitrogen chemicals,Xi'an 710065,China

[5] School of pharmacy, Xi'an medical university; hehao363@163.com (H.H.)

* Correspondence: tanghaifeng71@163.com; Tel.: 029-84774748 (H.T.);

blezhang@fmmu.edu.cn; Tel.: 029-84711513 (B.Z.);

Contents

NMR (1D and 2D) and MS spectra of compounds 1–5

Figure S1. Positive HR-ESI-MS spectrum of compound 1

Figure S2. ESI-MS spectrum of compound 1

Figure S3. ^{1}H NMR (800 MHz, CD_3OD) spectrum of compound 1

Figure S4. ^{13}C NMR (201 MHz, CD_3OD) spectrum of compound 1

Figure S5. DEPT 90 spectrum of Compound 1

Figure S6. DEPT 135 spectrum of compound **1**

Figure S7. HSQC spectrum of compound **1**

Figure S8. HMBC spectrum of compound **1**

Figure S9. ^{1}H-^{1}H COSY spectrum of compound **1**

Figure S10. NOESY spectrum of compound **1**

Figure S11. TOCSY spectrum of compound **1**

Figure S12. Positive HR-ESI-MS spectrum of compound **2**

Figure S13. ESI-MS spectrum of compound **2**

Figure S14. ^{1}H NMR (800 MHz, CD_3OD) spectrum of compound **2**

Figure S15. ^{13}C NMR (201 MHz, CD_3OD) spectrum of compound **2**

Figure S16. DEPT 90 spectrum of Compound **2**

Figure S17. DEPT 135 spectrum of compound **2**

Figure S18. HSQC spectrum of compound **2**

Figure S19. HMBC spectrum of compound **2**

Figure S20. ^{1}H-^{1}H COSY spectrum of compound **2**

Figure S21. NOESY spectrum of compound **2**

Figure S22. TOCSY spectrum of compound **2**

Figure S23. Positive HR-ESI-MS spectrum of compound **3**

Figure S24. ESI-MS spectrum of compound **3**

Figure S25. ^{1}H NMR (800 MHz, CD_3OD) spectrum of compound **3**

Figure S26. ^{13}C NMR (201 MHz, CD_3OD) spectrum of compound **3**

Figure S27. DEPT 90 spectrum of compound **3**

Figure S28. DEPT 135 spectrum of compound **3**

Figure S29. HSQC spectrum of compound **3**

Figure S30. HMBC spectrum of compound **3**

Figure S31. ^{1}H-^{1}H COSY spectrum of compound **3**

Figure S32. NOESY spectrum of compound **3**

Figure S33. TOCSY spectrum of compound **3**

Figure S34. Positive HR-ESI-MS spectrum of compound **4**

Figure S35. ESI-MS spectrum of compound **4**

Figure S36. ^{1}H NMR (800 MHz, CD_3OD) spectrum of compound **4**

Figure S37. ^{13}C NMR (201 MHz, CD_3OD) spectrum of compound **4**

Figure S38. DEPT 90 spectrum of compound **4**

Figure S39. DEPT 135 spectrum of compound **4**

Figure S40. HSQC spectrum of compound **4**

Figure S41. HMBC spectrum of compound **4**

Figure S42. ^1H-^1H COSY spectrum of compound **4**

Figure S43. NOESY spectrum of compound **4**

Figure S44. TOCSY spectrum of compound **4**

Figure S45. Positive HR-ESI-MS spectrum of compound **5**

Figure S46. ESI-MS spectrum of compound **5**

Figure S47. ^1H NMR (800 MHz, CD_3OD) spectrum of compound **5**

Figure S48. ^{13}C NMR (201 MHz, CD_3OD) spectrum of compound **5**

Figure S49. DEPT 90 spectrum of compound **5**

Figure S50. DEPT 135 spectrum of compound **5**

Figure S51. HSQC spectrum of compound **5**

Figure S52. HMBC spectrum of compound **5**

Figure S53. ^1H-^1H COSY spectrum of compound **5**

Figure S54. NOESY spectrum of compound **5**

Figure S55. TOCSY spectrum of compound **5**

Figure 1. Positive HR-ESI-MS spectrum of Compound **1.**

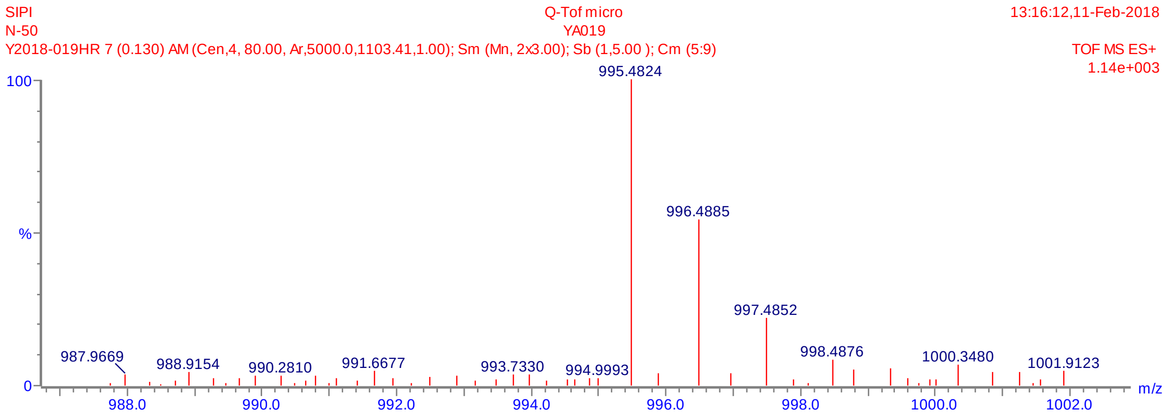

Figure S2. ESI-MS spectrum of Compound **1**

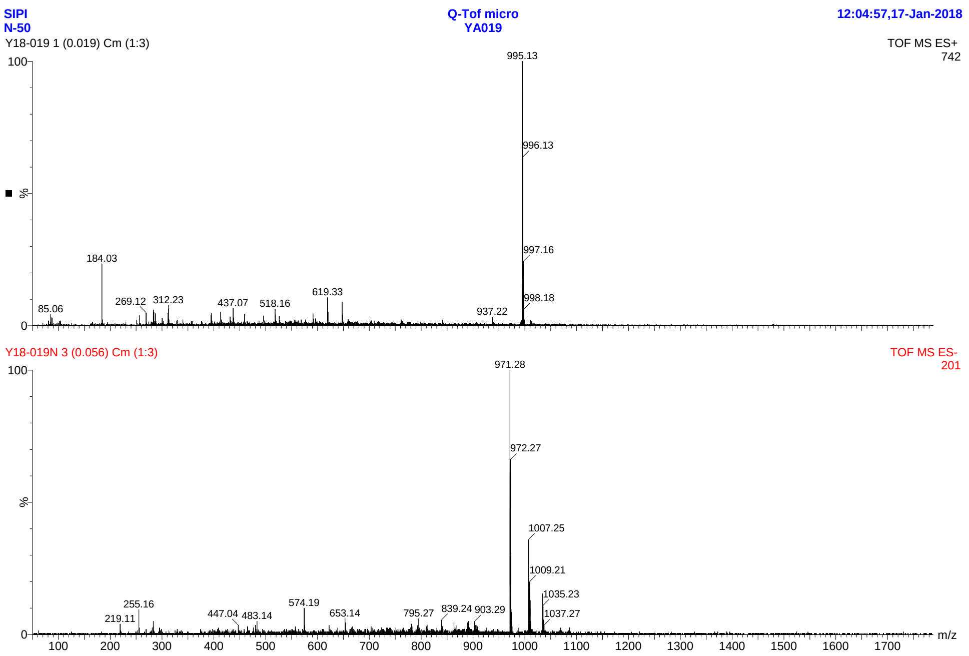

Figure S3. ^{1}H-NMR spectrum of Compound **1**

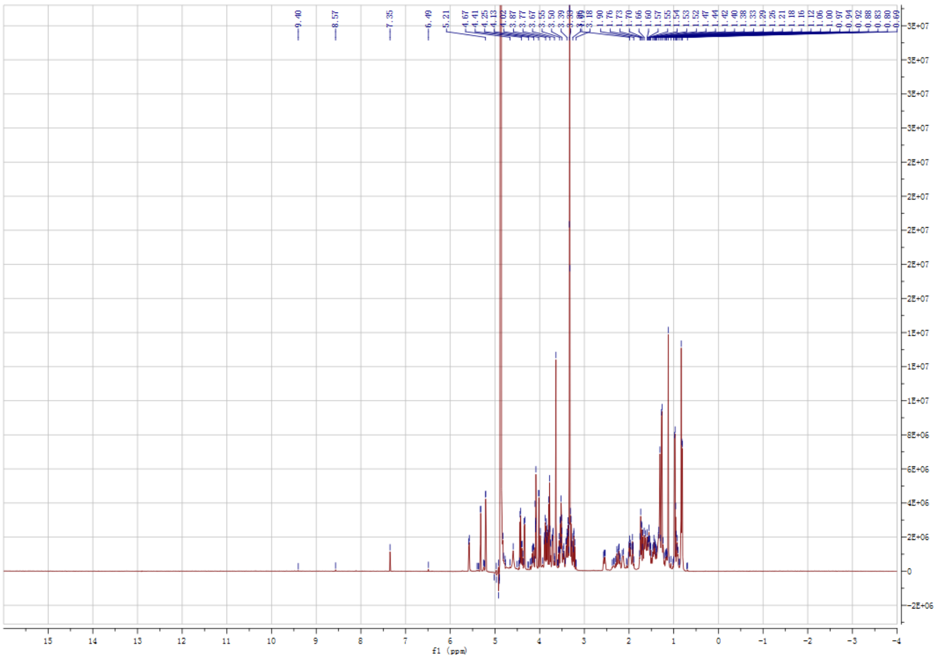

Figure S4. ^{13}C NMR (201 MHz, CD_3OD) spectrum of compound **1**

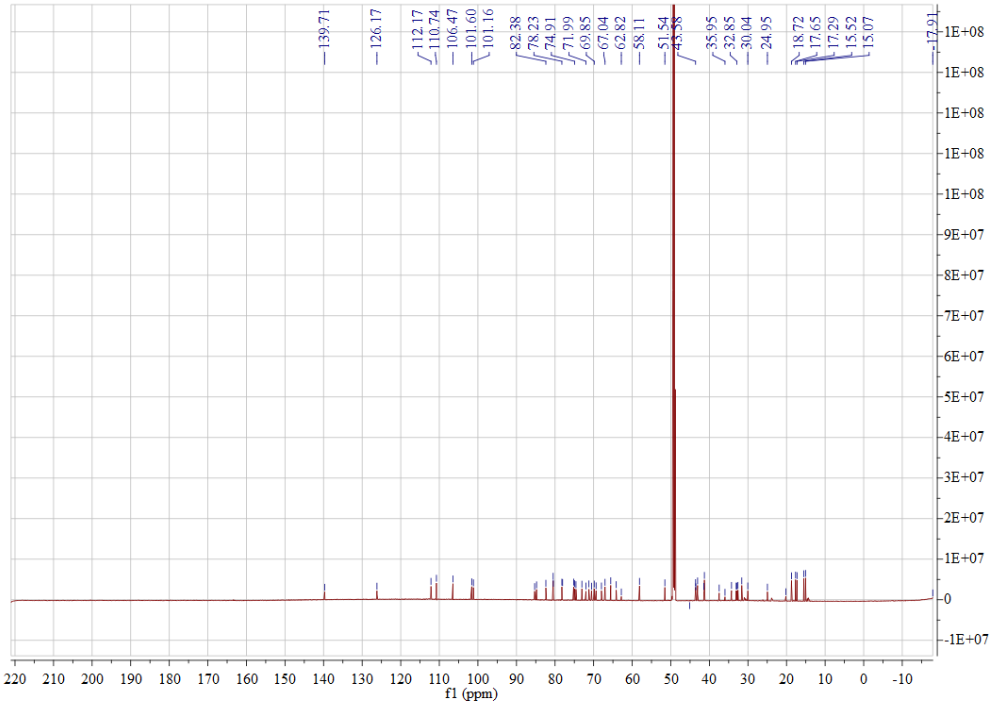

Figure S5. DEPT 90 spectrum of Compound **1**

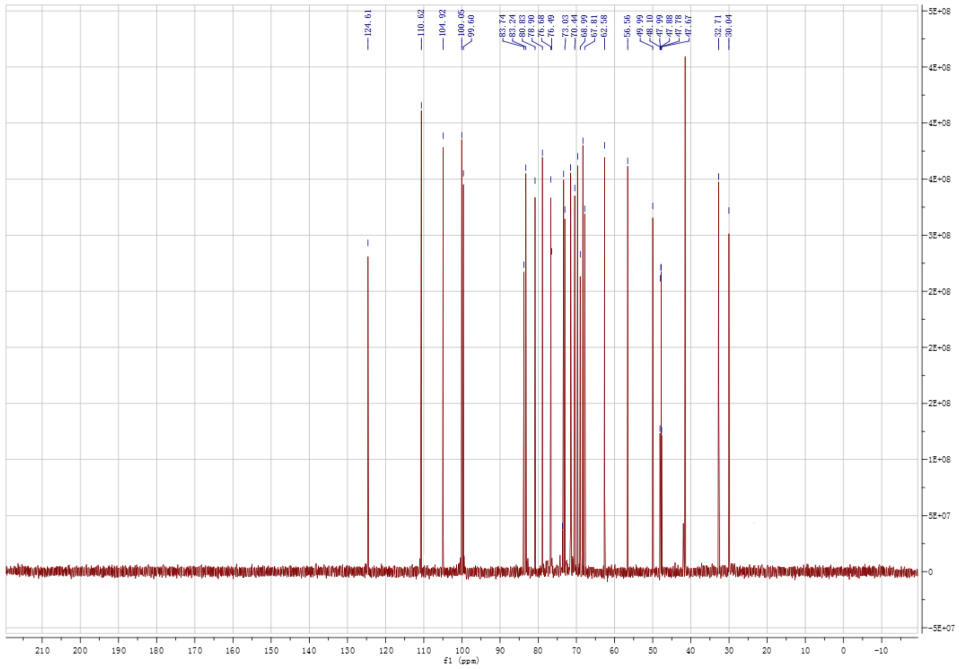

Figure S6. DEPT 135 spectrum of Compound **1**

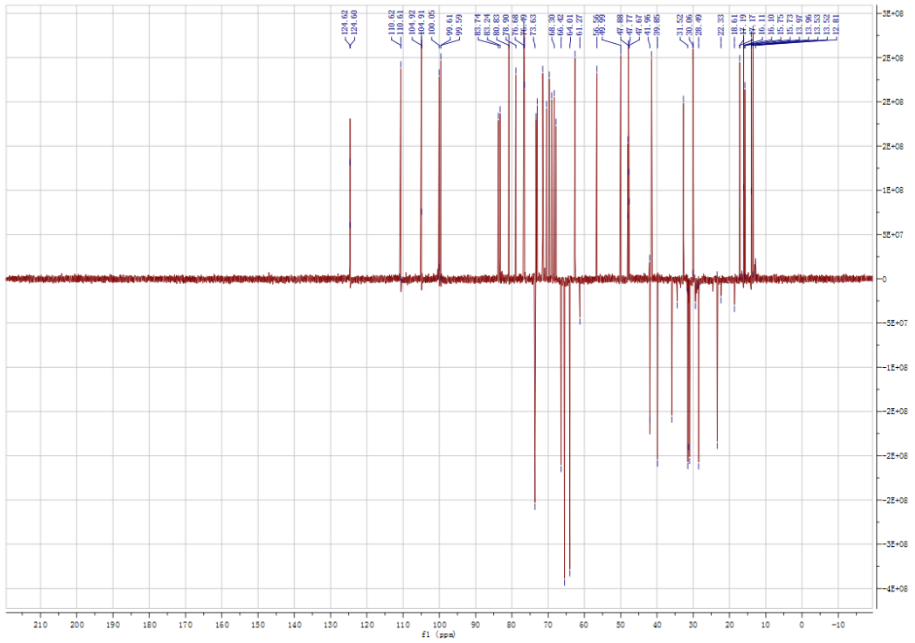

Figure S7. HSQC spectrum of Compound **1**

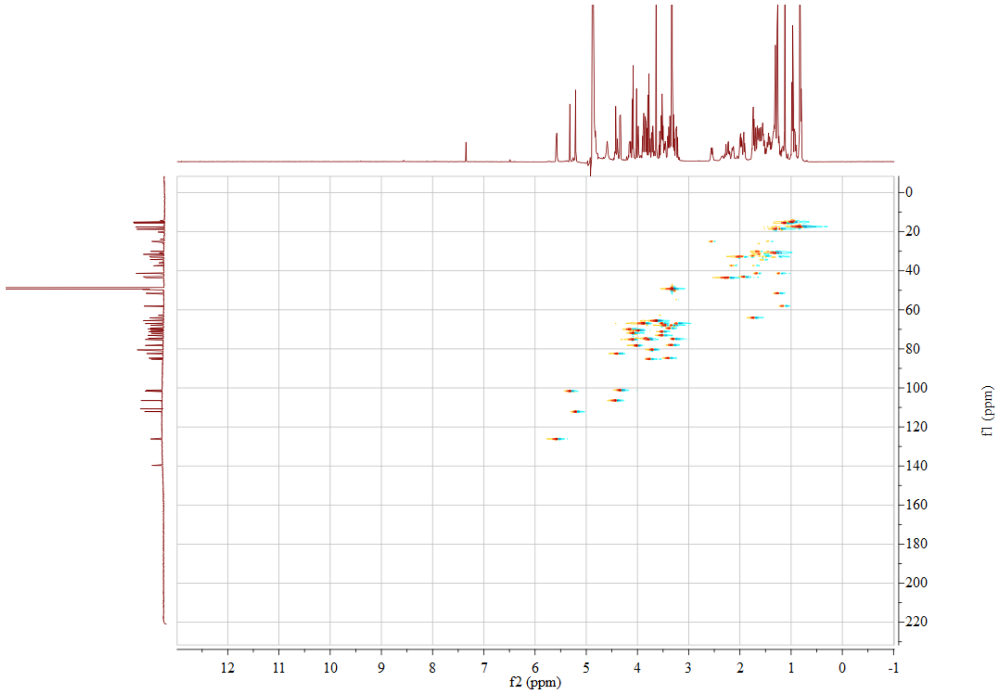

Figure S8. HMBC spectrum of Compound **1**

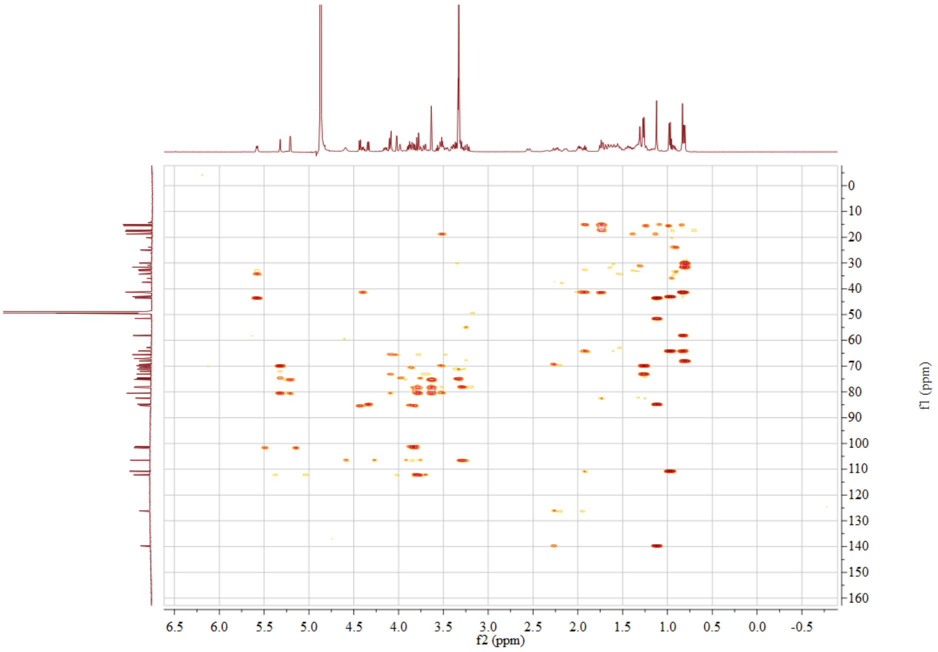

Figure S9. ^{1}H-^{1}H COSY spectrum of Compound **1**

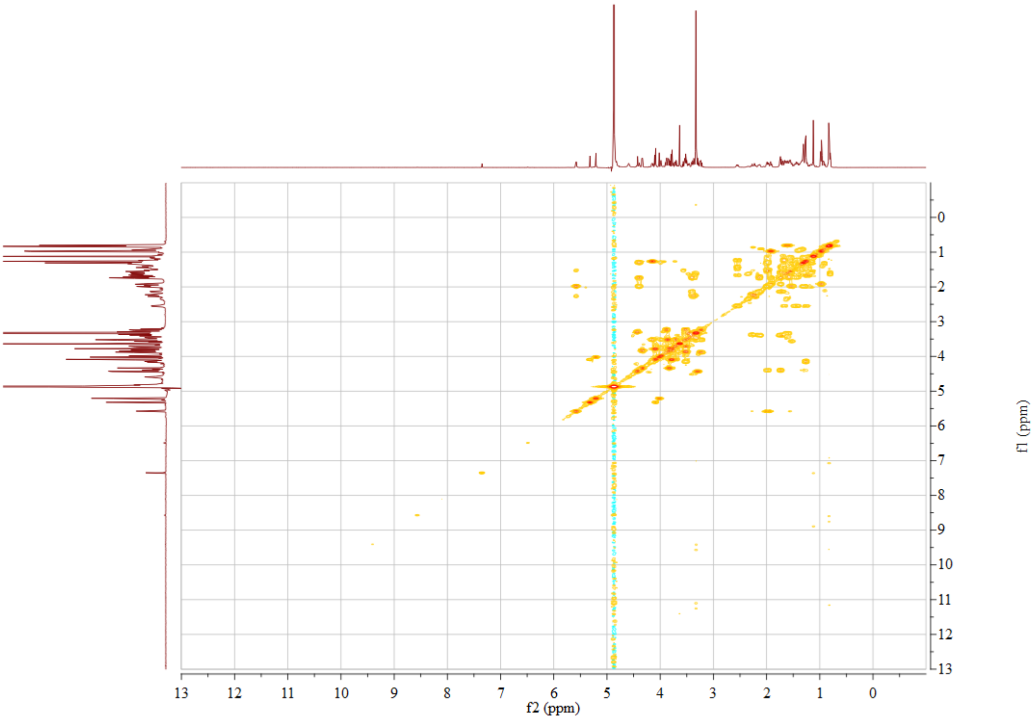

Figure S10. NOESY spectrum of Compound **1**

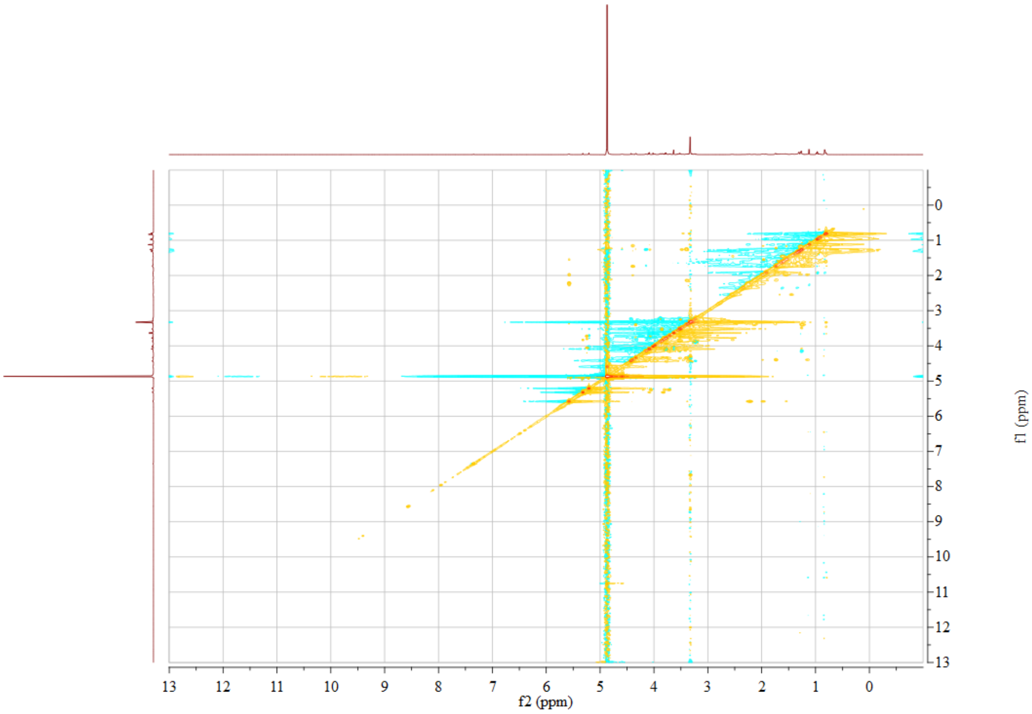

Figure S11. TOCSY spectrum of compound **1**

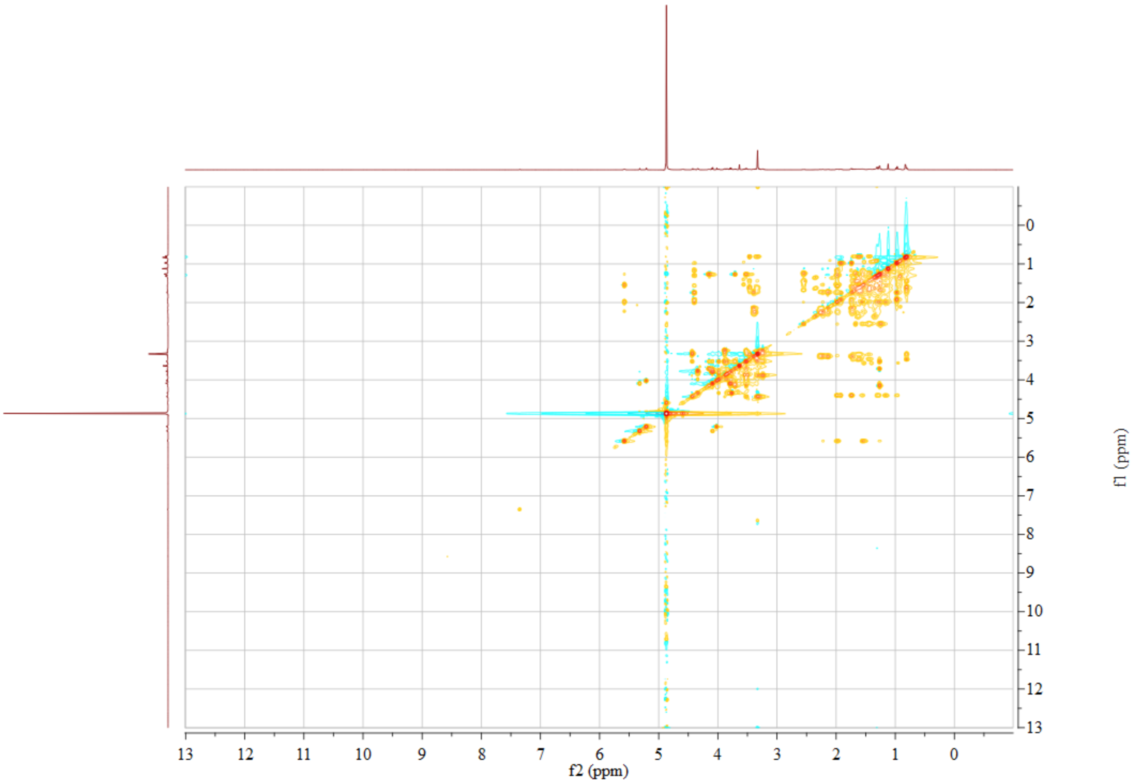

Figure S12. Positive HR-ESI-MS spectrum of Compound **2**

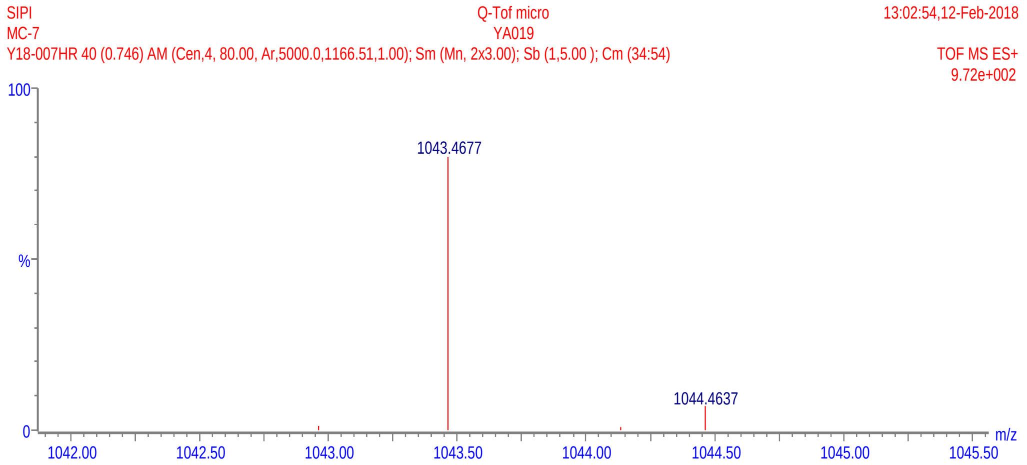

Figure S13. Positive ESI-MS spectrum of Compound **2**

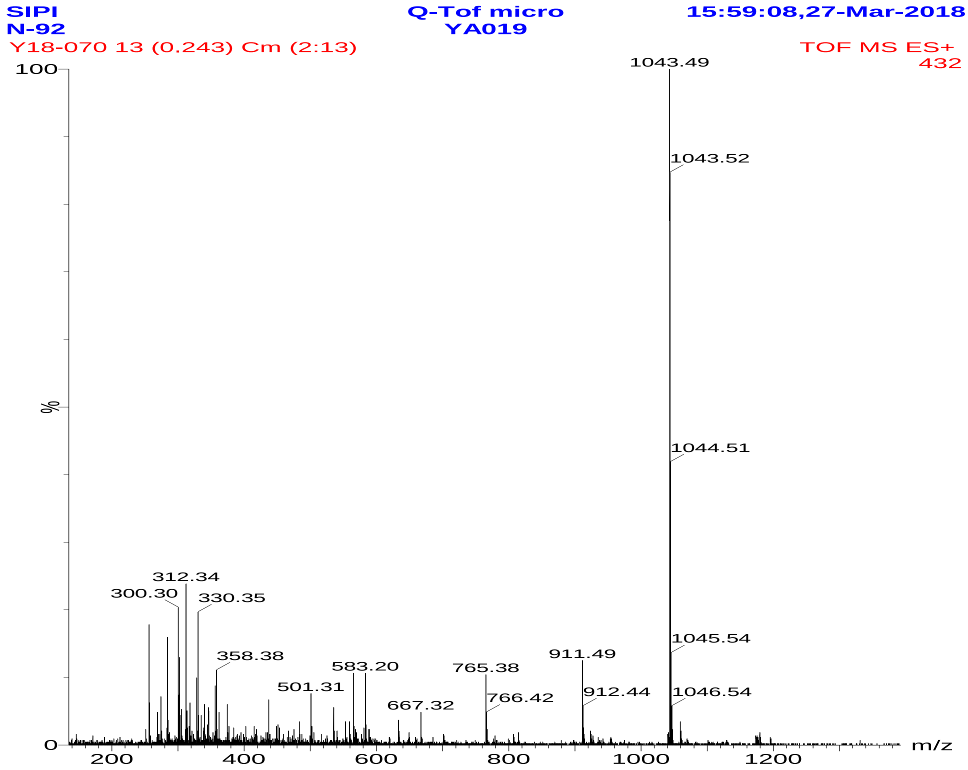

Figure S14. ^{1}H-NMR spectrum of Compound **2**

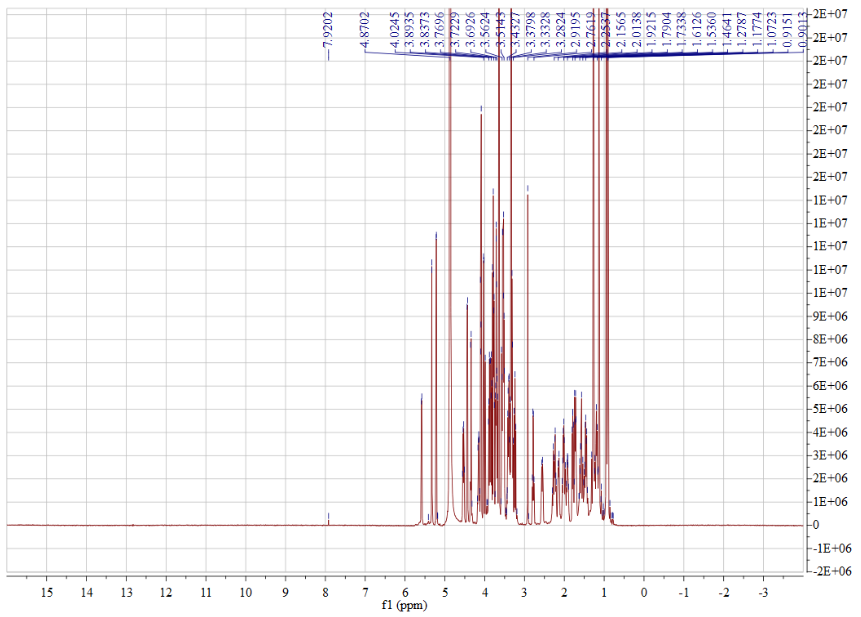

Figure S15. ^{13}C-NMR spectrum of Compound **2**

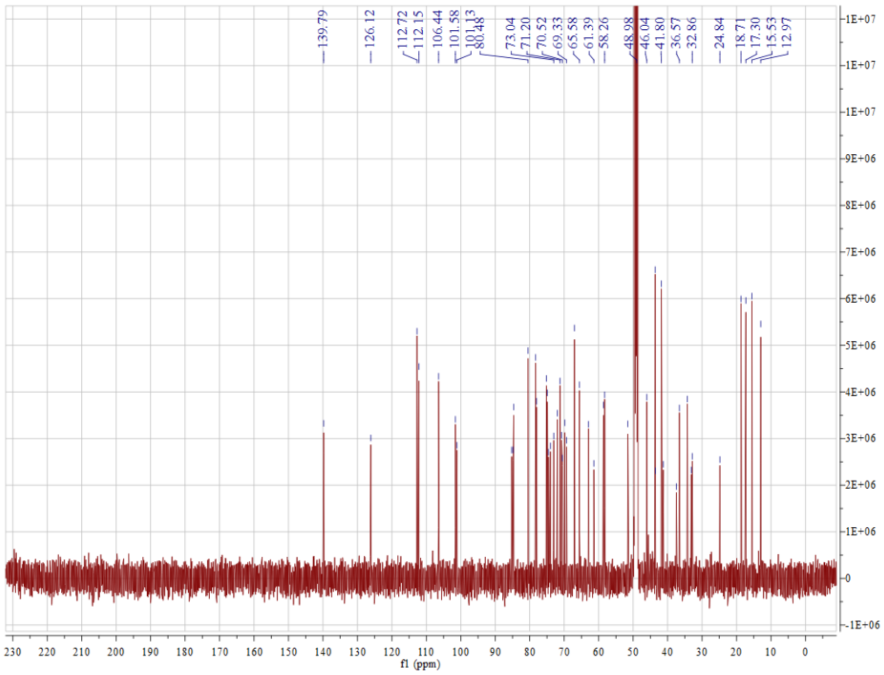

Figure S16. DEPT 90 spectrum of Compound **2**

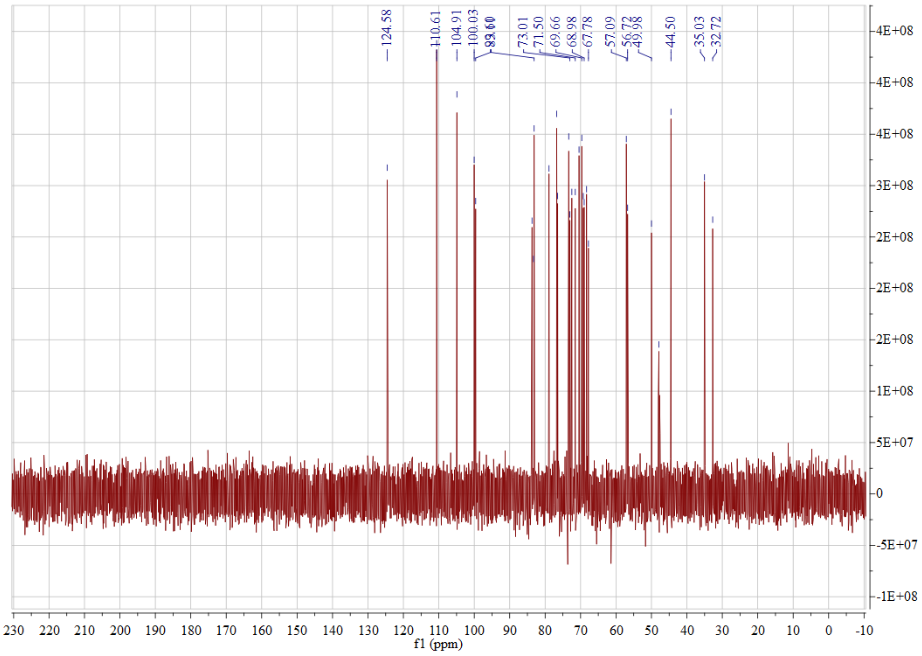

Figure S17. DEPT 135 spectrum of Compound **2**

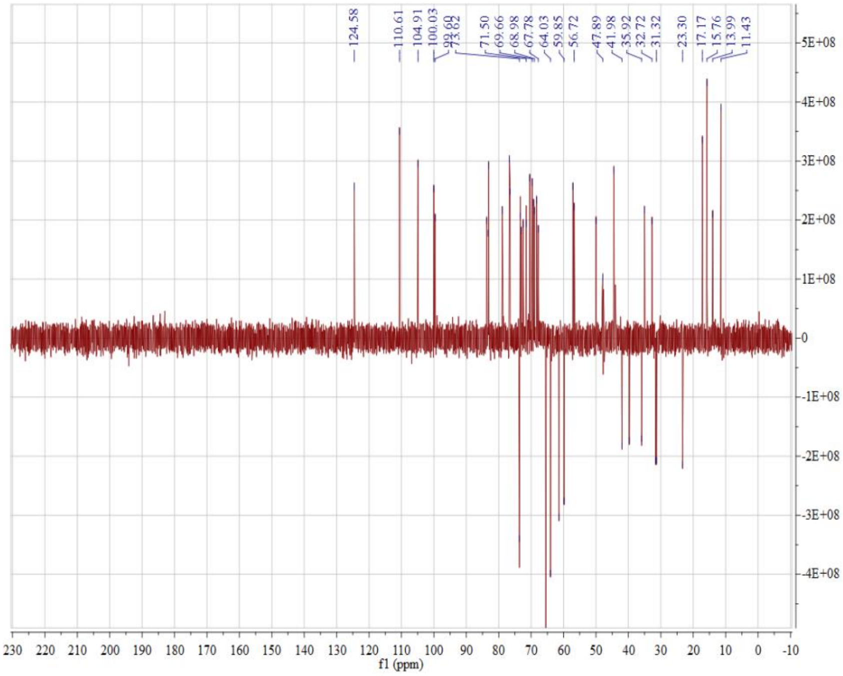

Figure S18. HSQC spectrum of Compound **2**

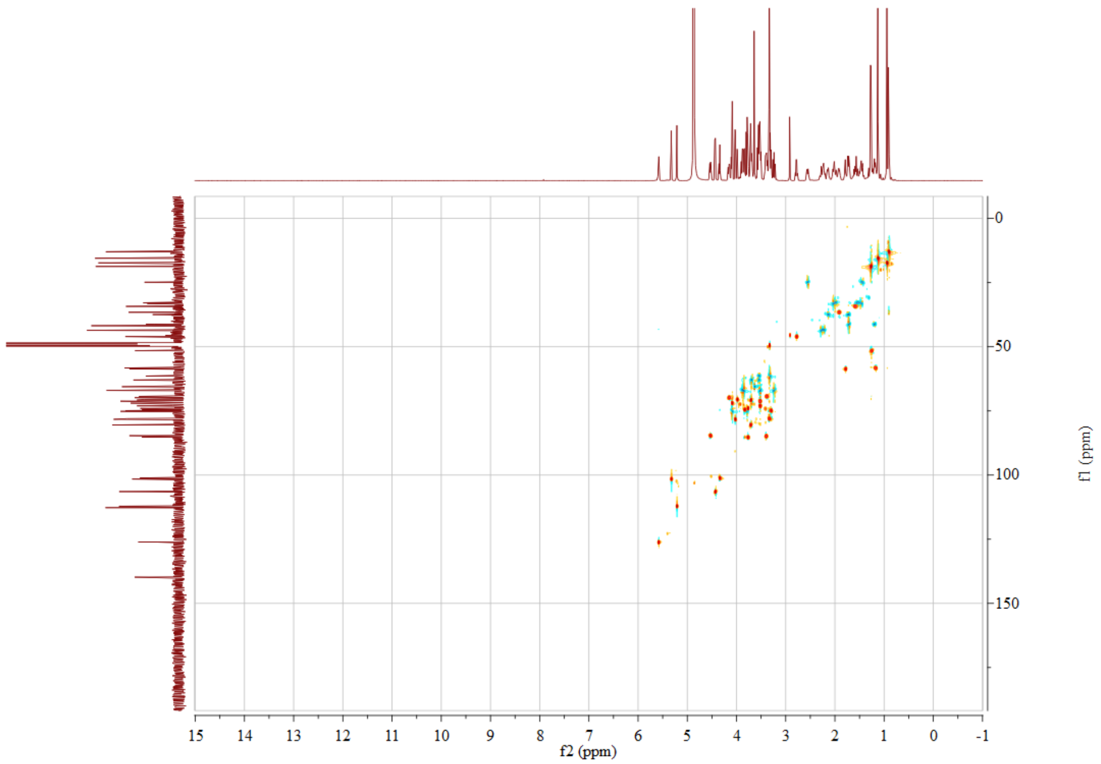

Figure S19. HMBC spectrum of Compound **2**

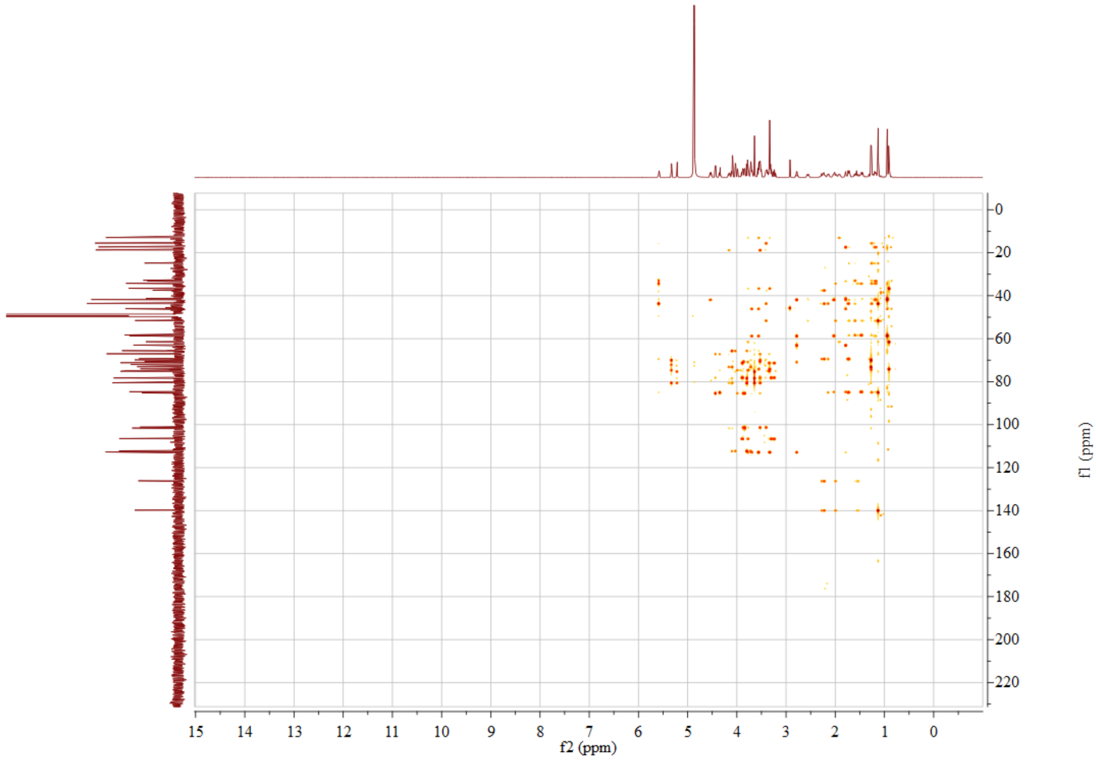

Figure S20. ^{1}H-^{1}H COSY spectrum of Compound **2**

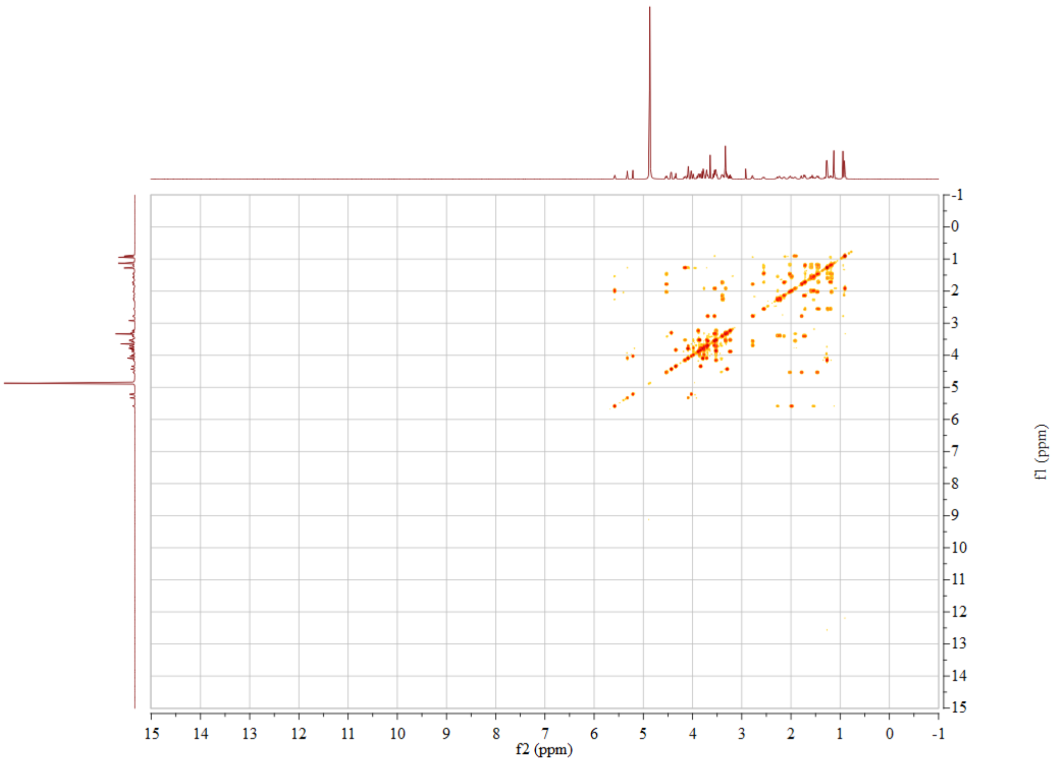

Figure S21. NOESY spectrum of Compound **2**

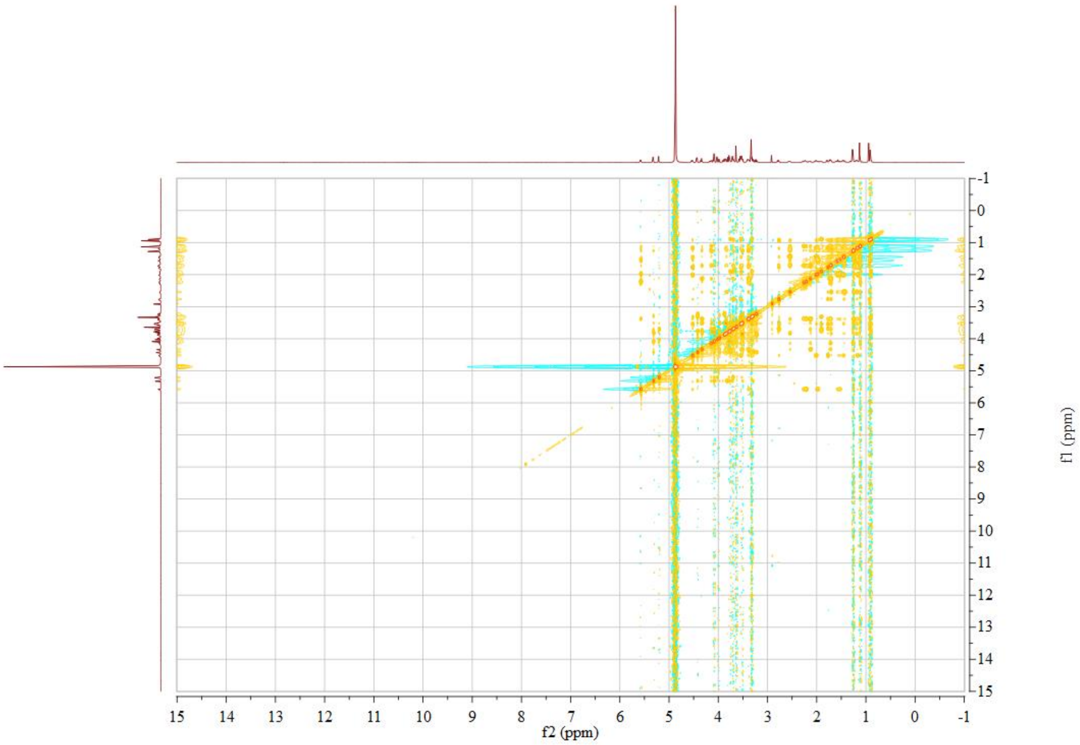

Figure S22. TOCSY spectrum of Compound **2**

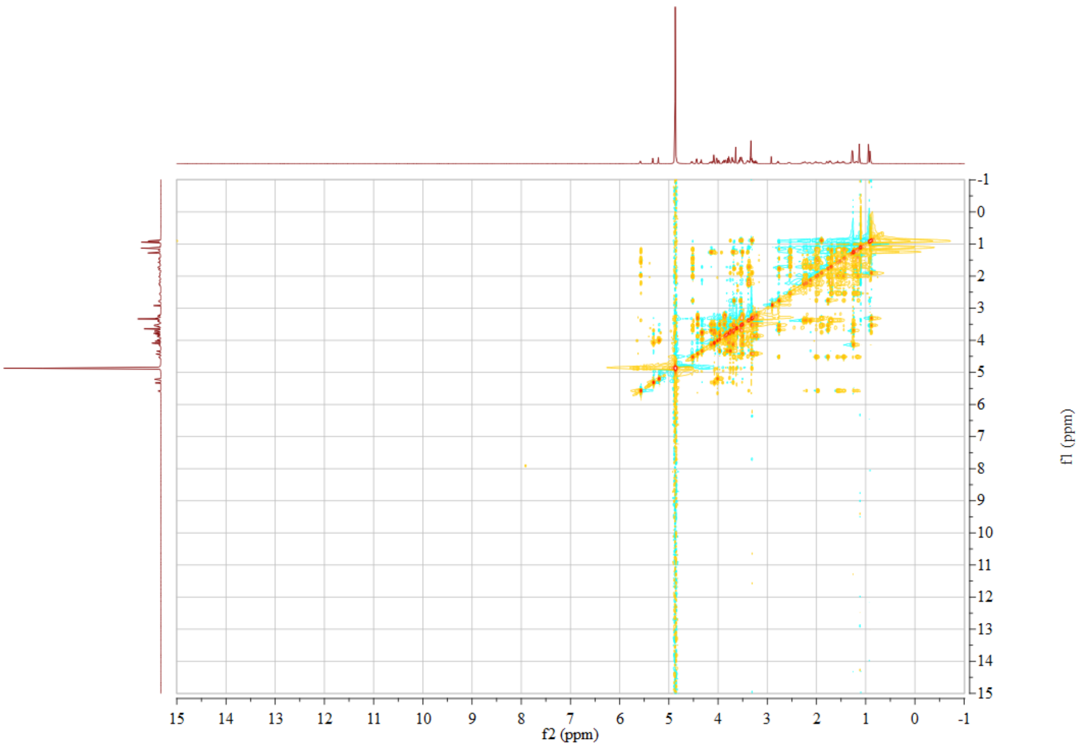

Figure S23. Positive HR-ESI-MS spectrum of Compound **3**

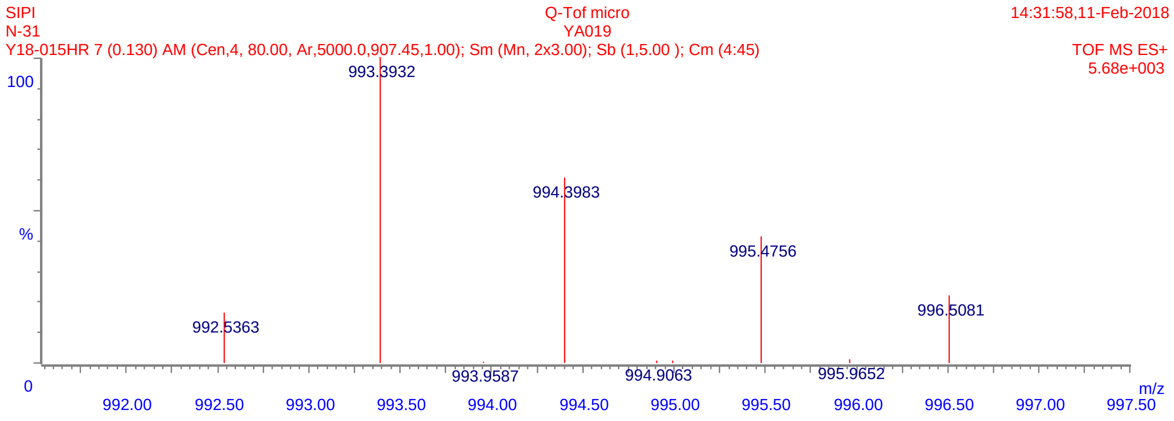

Figure S24. ESI-MS spectrum of Compound **3**

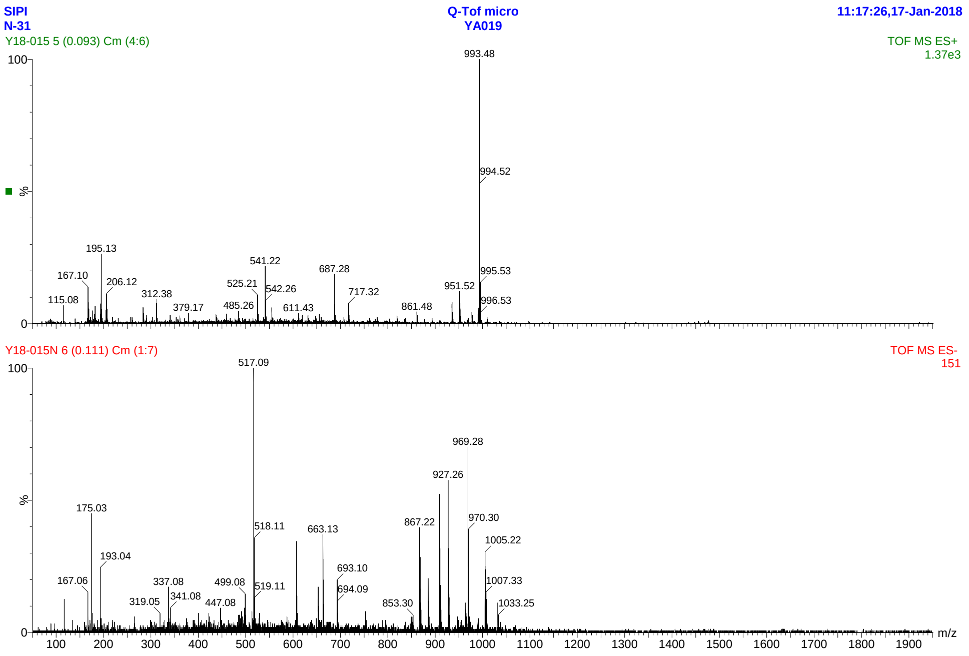

Figure S25.^{1}H-NMR spectrum of Compound **3**

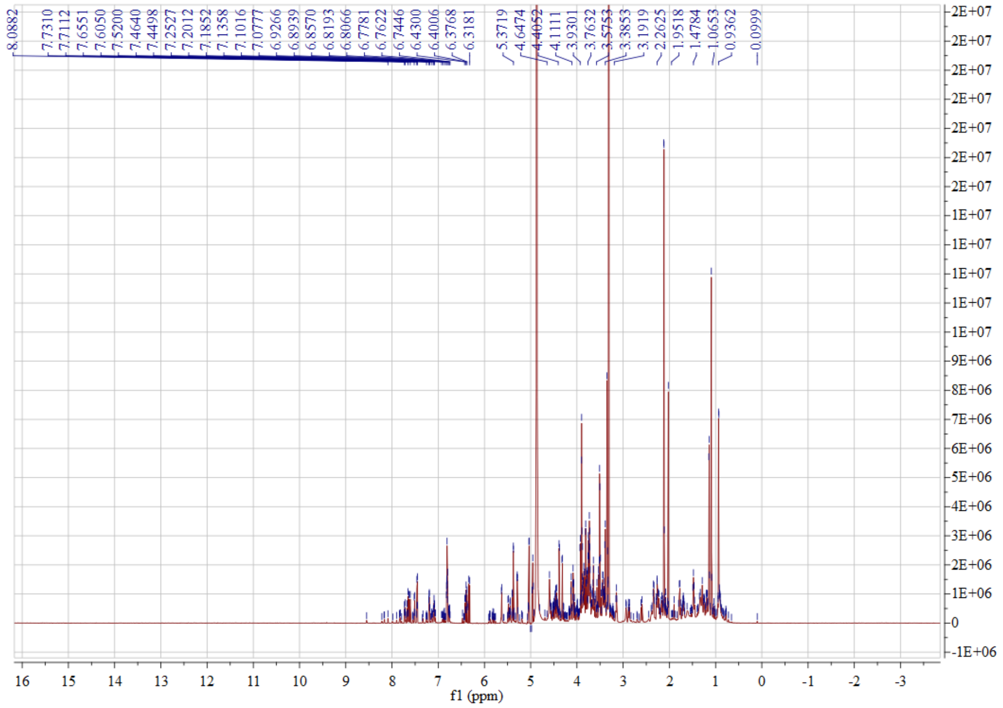

Figure S26.^{13}C-NMR spectrum of Compound **3**

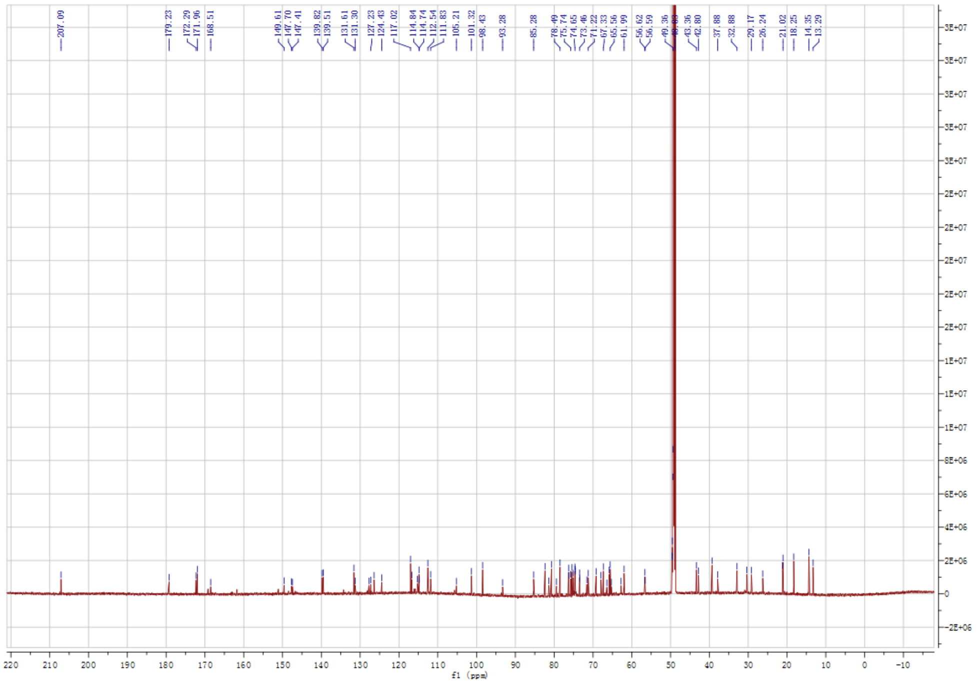

Figure S27. DEPT 90 spectrum of Compound **3**

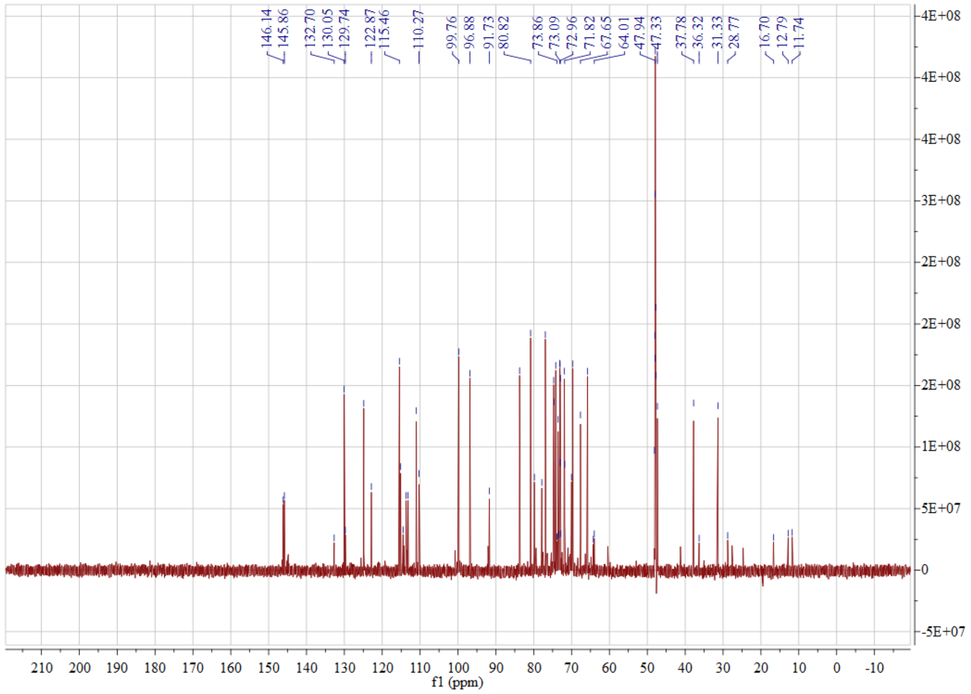

Figure S28. DEPT 135 spectrum of Compound **3**

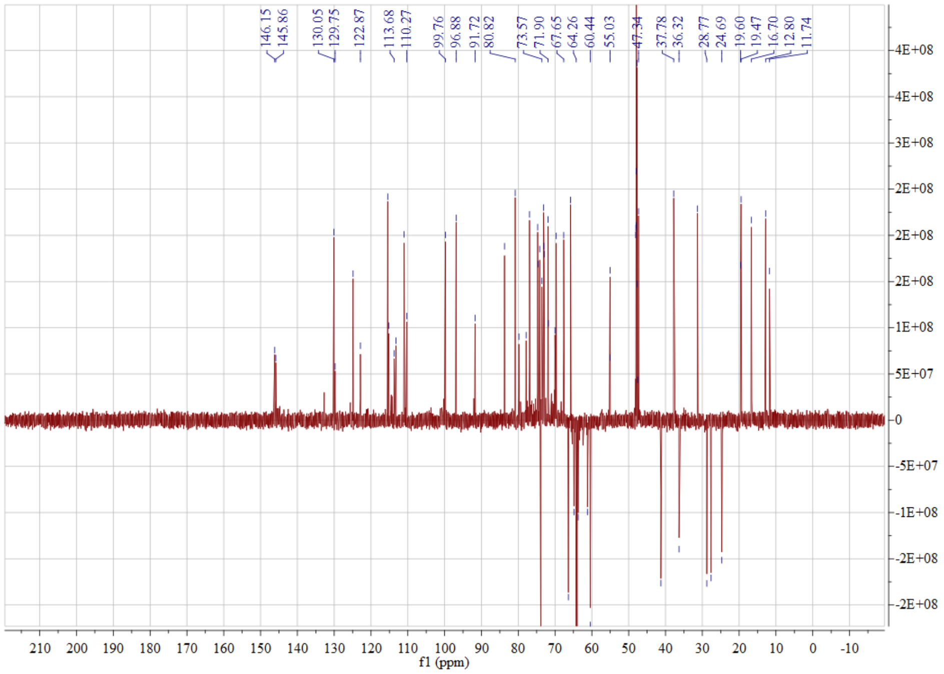

Figure S29. HSQC spectrum of Compound **3**

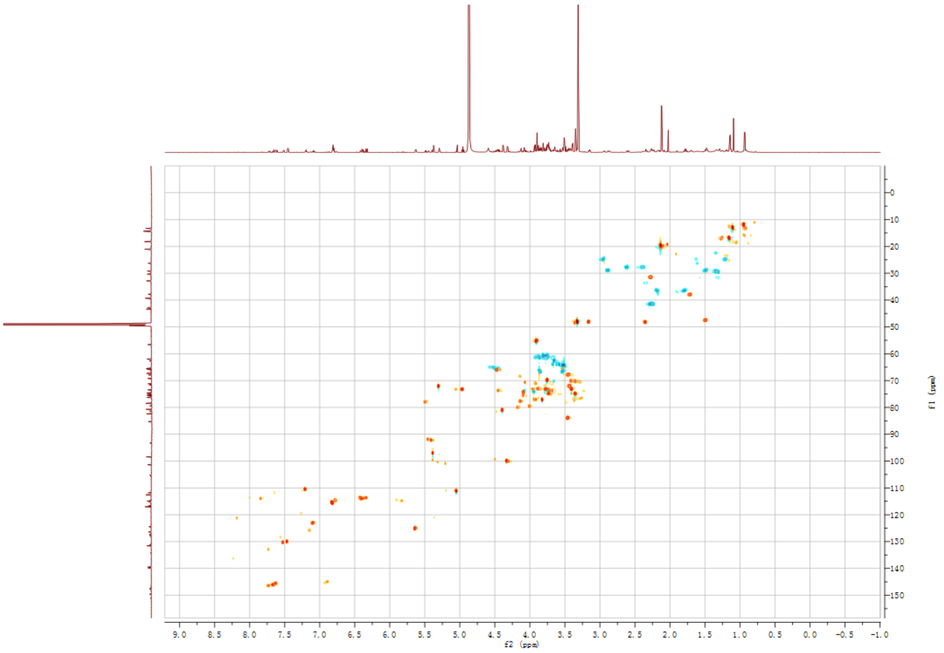

Figure S30. HMBC spectrum of Compound **3**

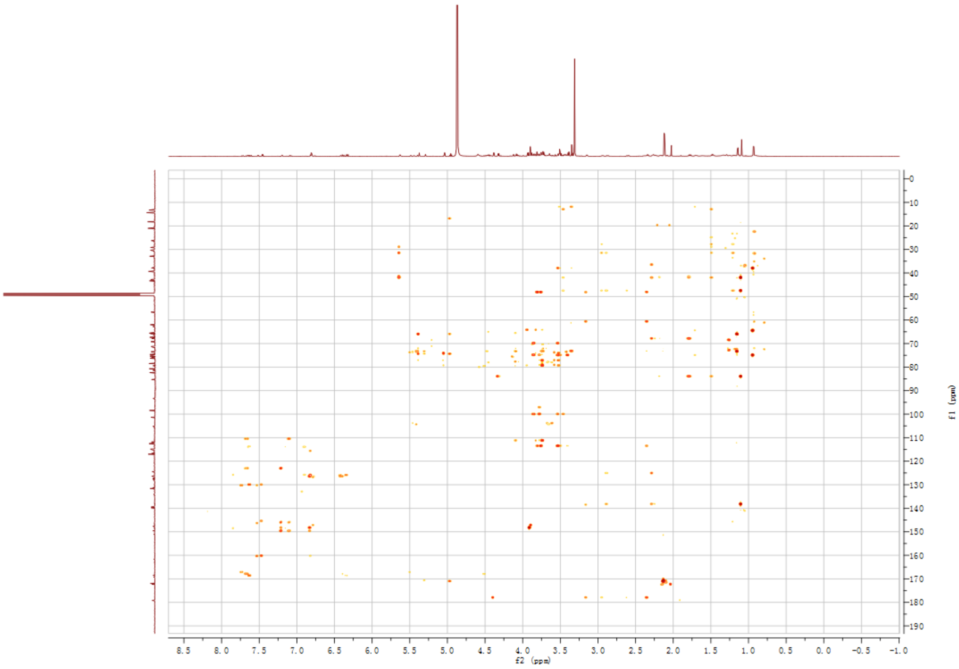

Figure S31. ^{1}H-^{1}H COSY spectrum of Compound **3**

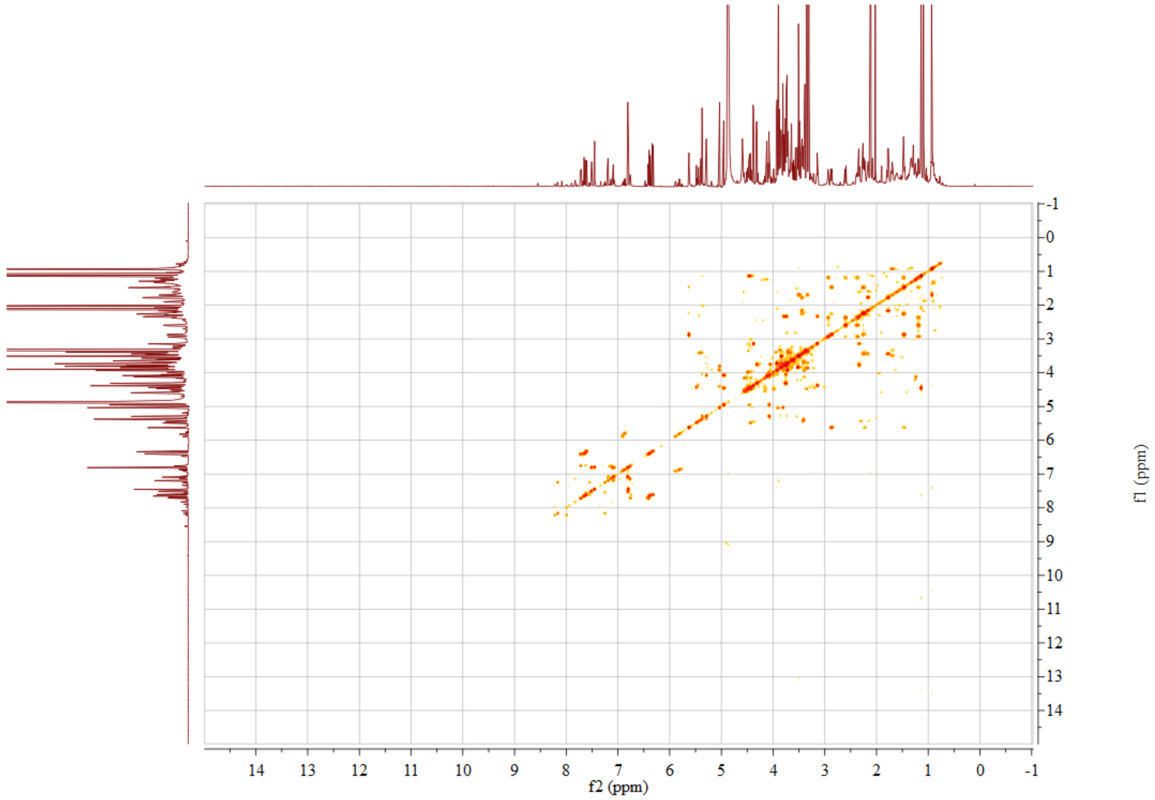

Figure S32. NOESY spectrum of Compound **3**

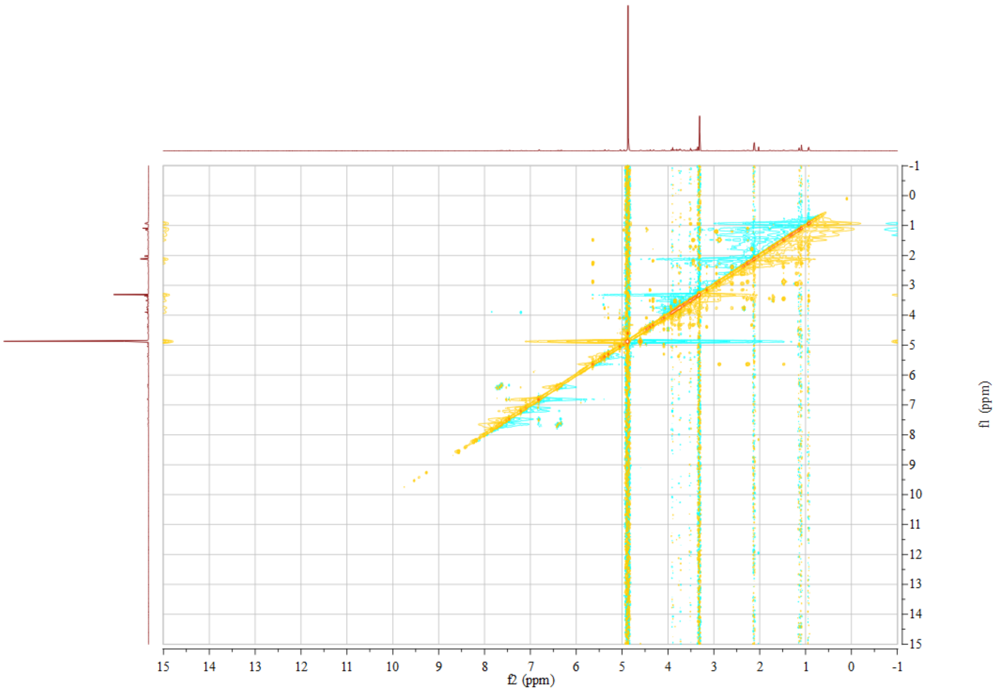

Figure S33. TOCSY spectrum of Compound **3**

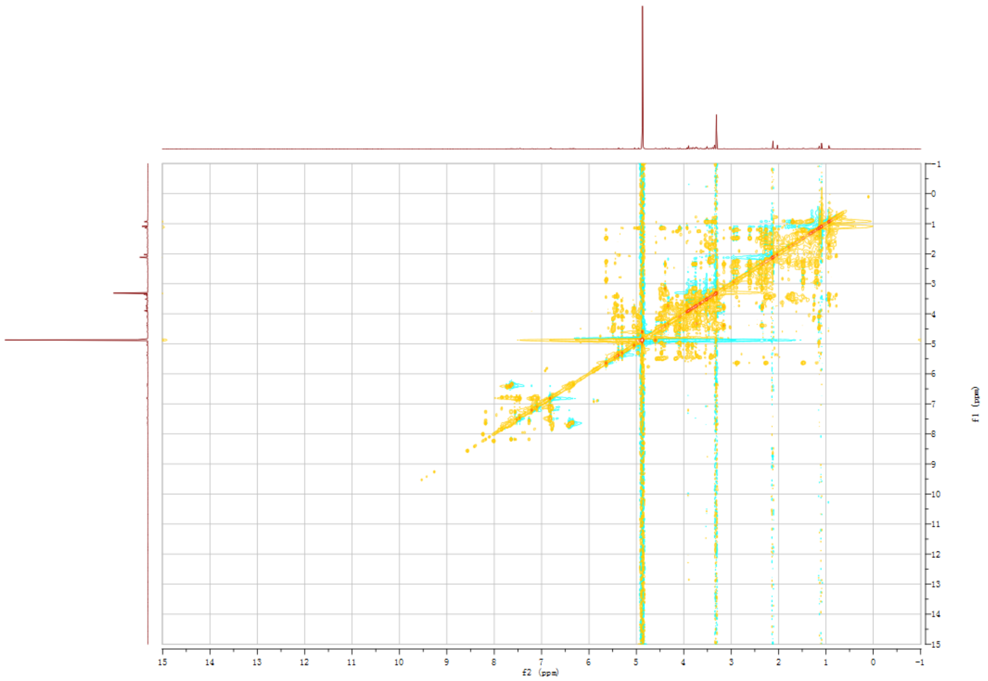

Figure S34. Positive HR-ESI-MS spectrum of Compound **4**

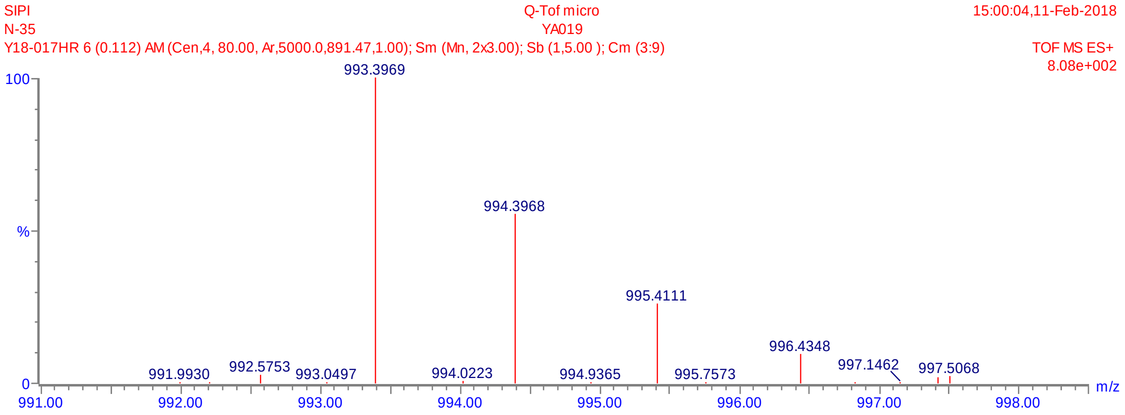

Figure S35. ESI-MS spectrum of Compound **4**

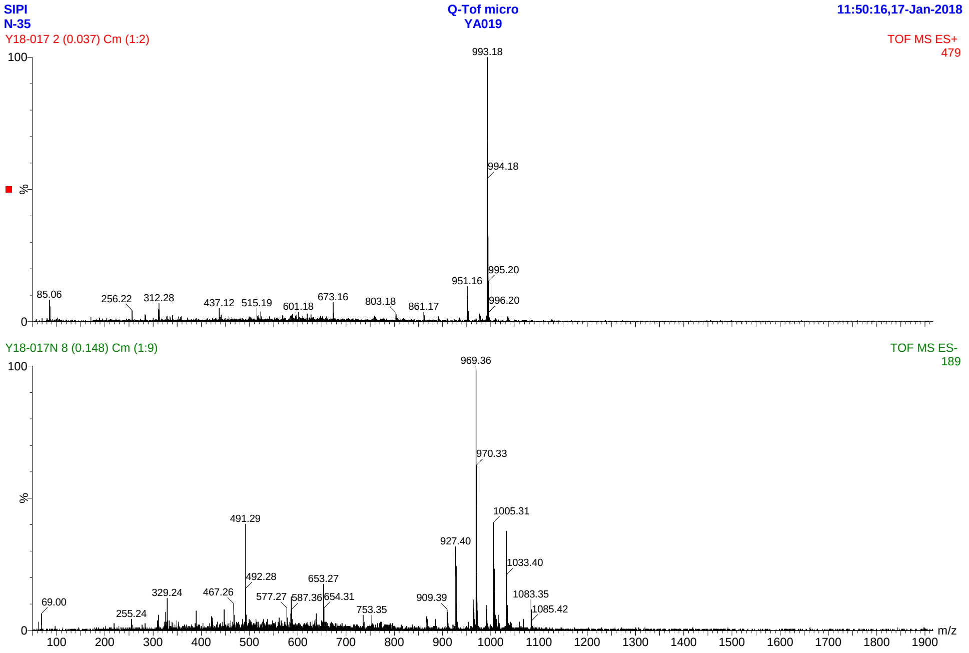

Figure S36. ^{1}H-NMR spectrum of Compound **4**

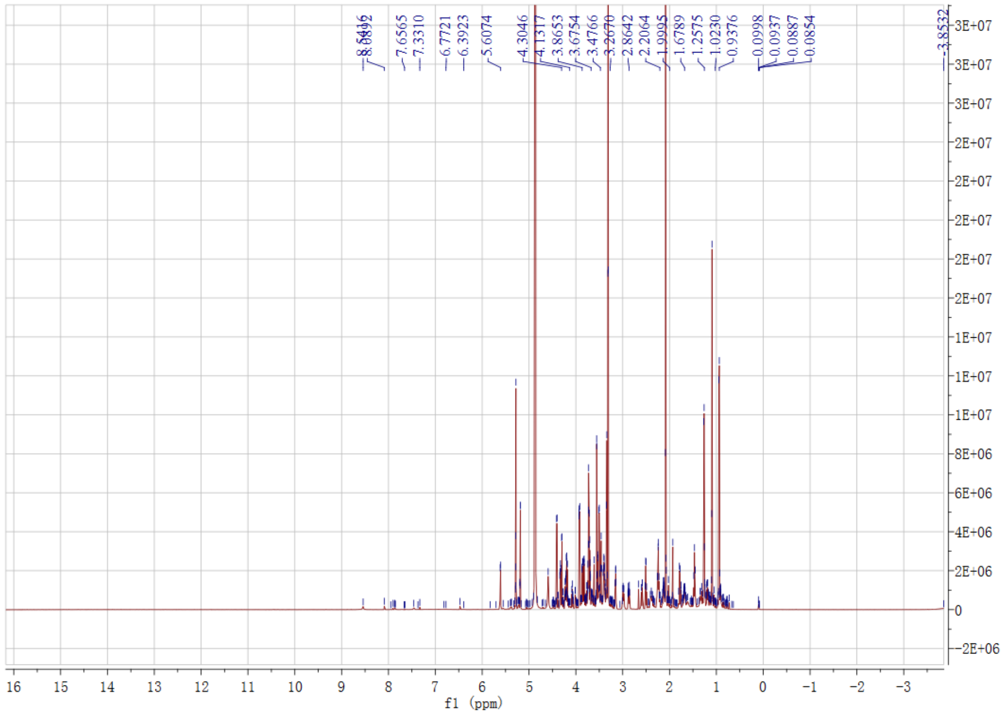

Figure S37. ^{13}C-NMR spectrum of Compound **4**

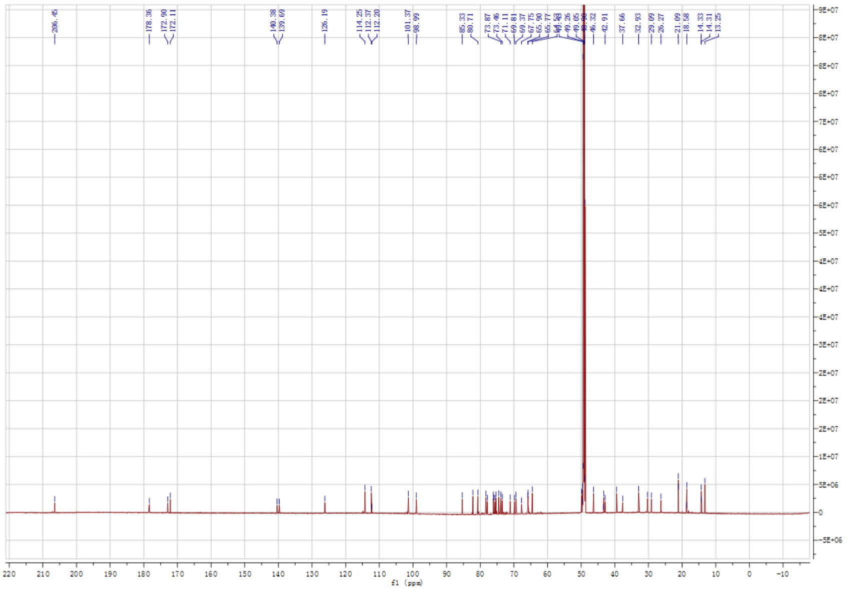

Figure S38. DEPT 90 spectrum of Compound **4**

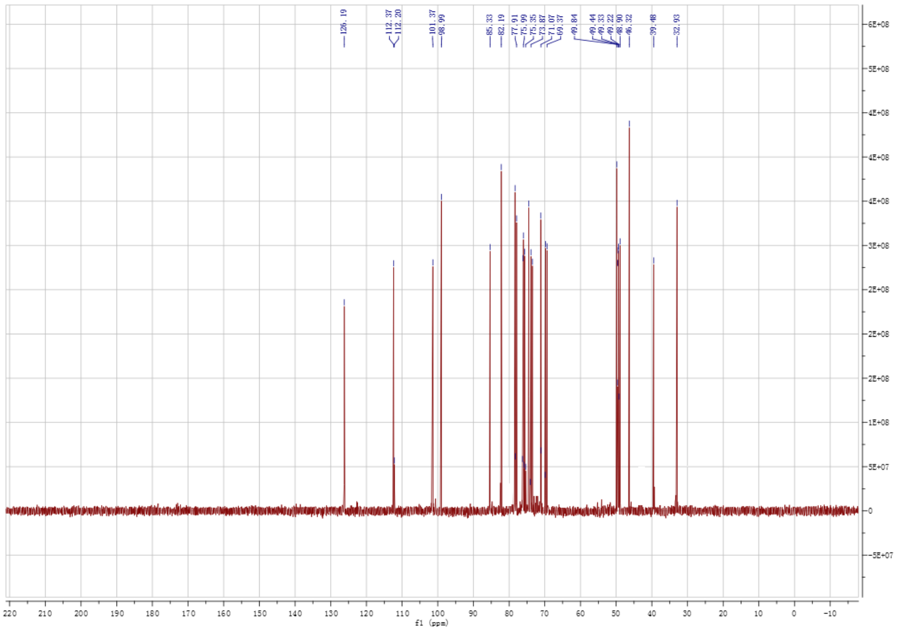

Figure S39. DEPT 135 spectrum of Compound **4**

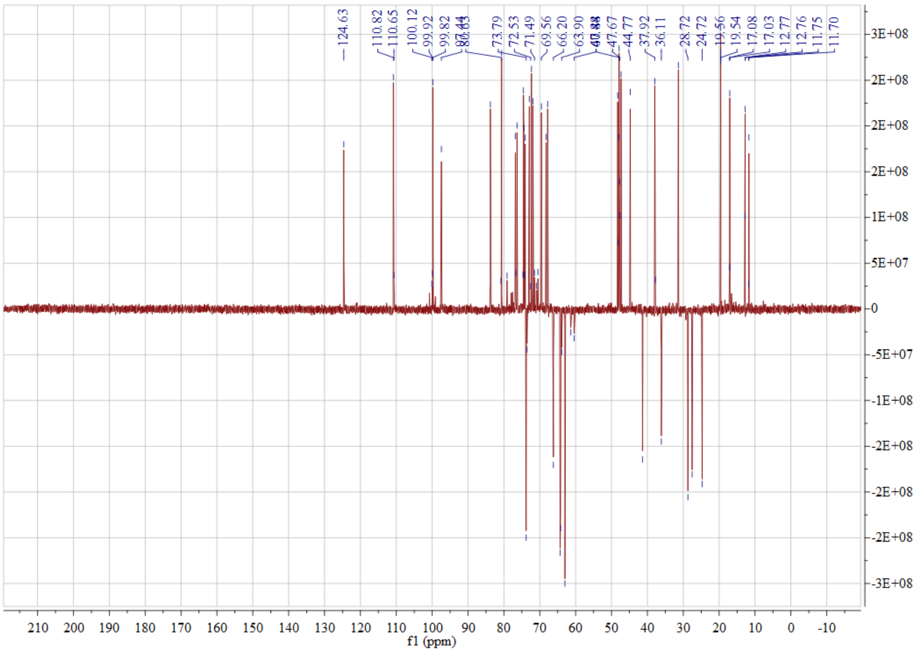

Figure S40. HSQC spectrum of Compound **4**

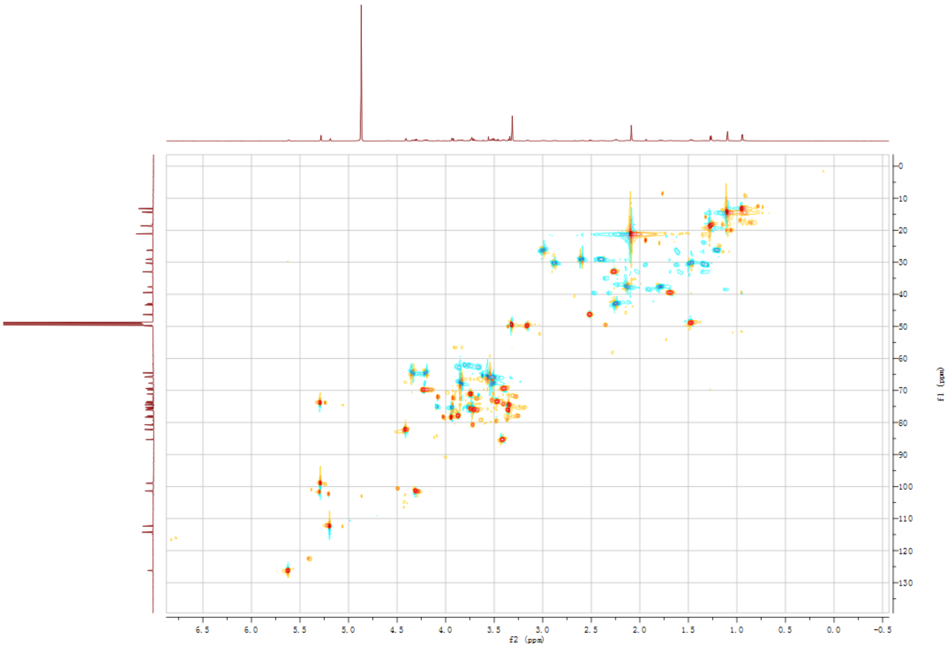

Figure S41. HMBC spectrum of Compound **4**

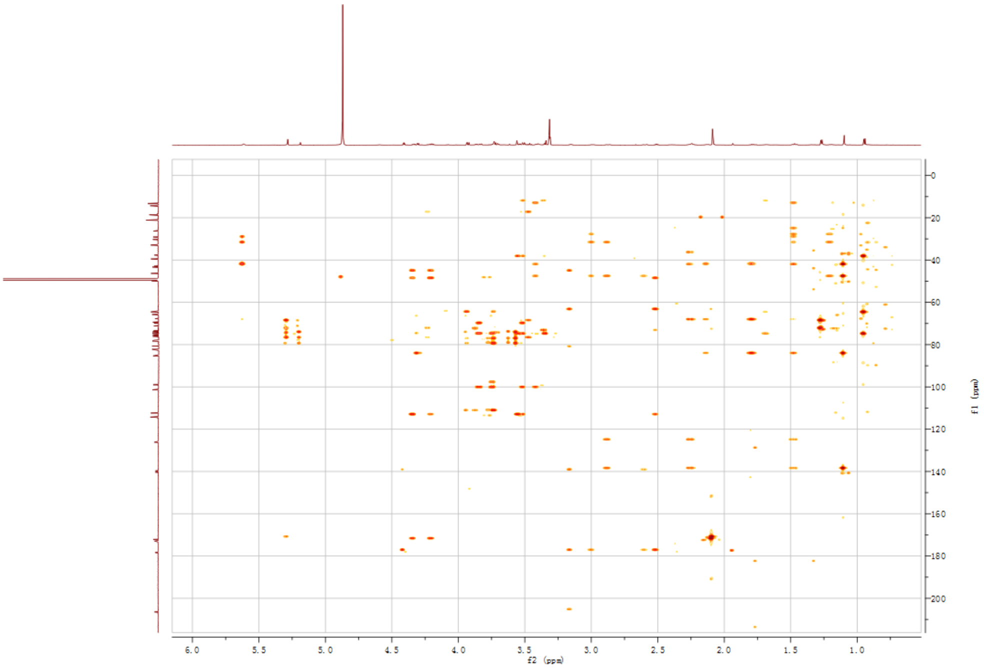

Figure S42. ^1H-^1H COSY spectrum of Compound **4**

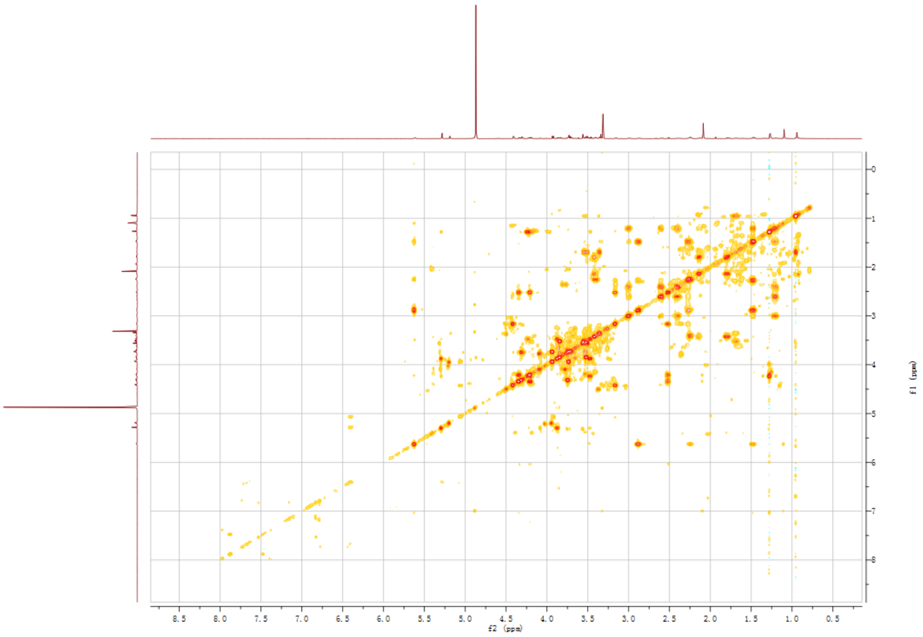

Figure S43. NOESY spectrum of Compound **4**

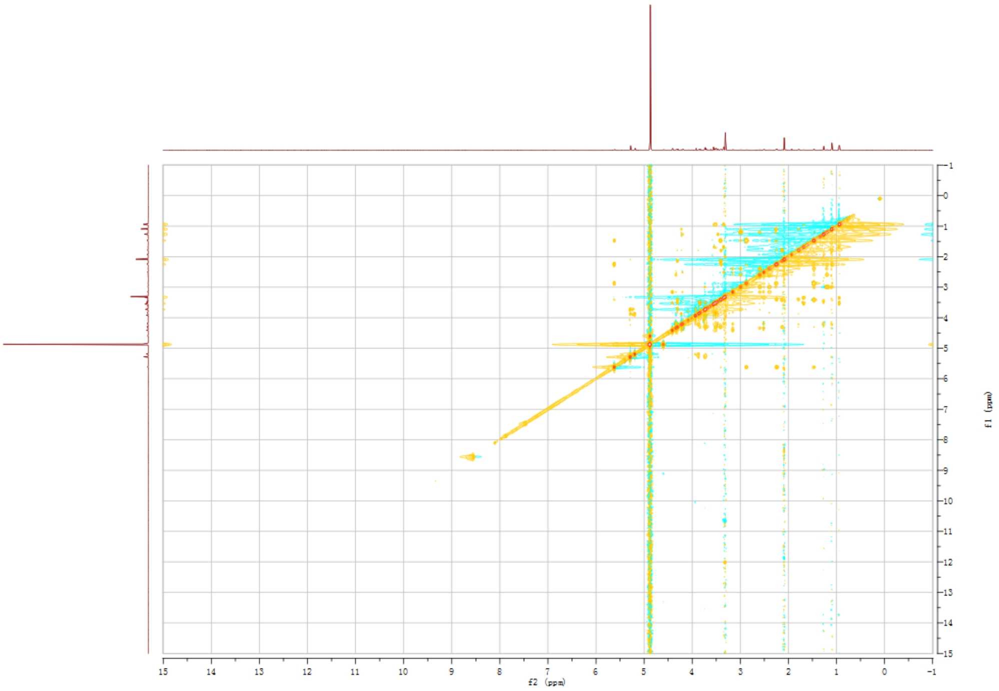

Figure S44. TOCSY spectrum of Compound **4**

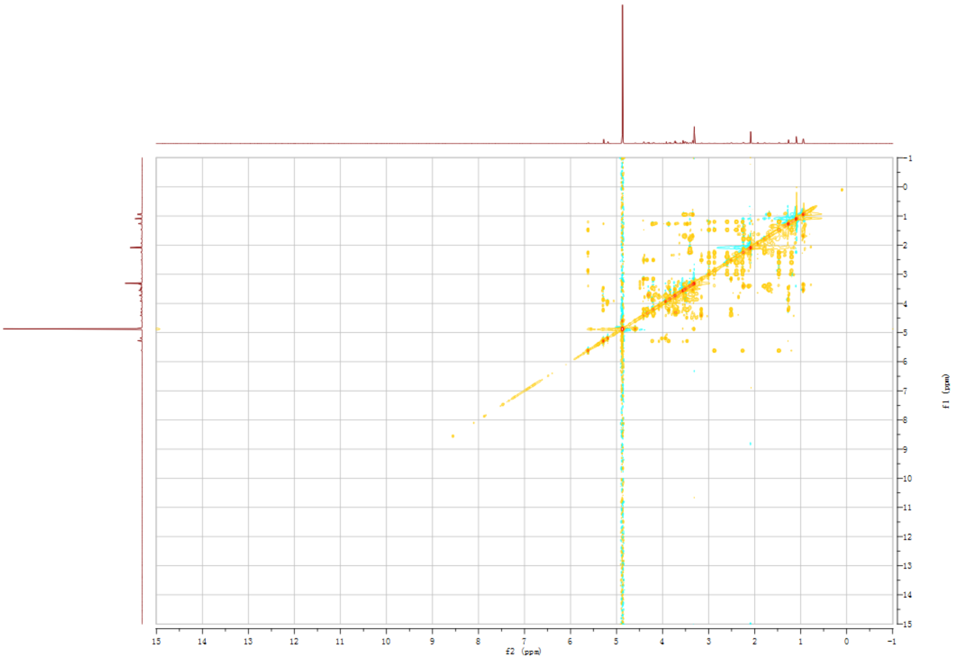

Figure S45. Positive HR-ESI-MS spectrum of Compound **5**

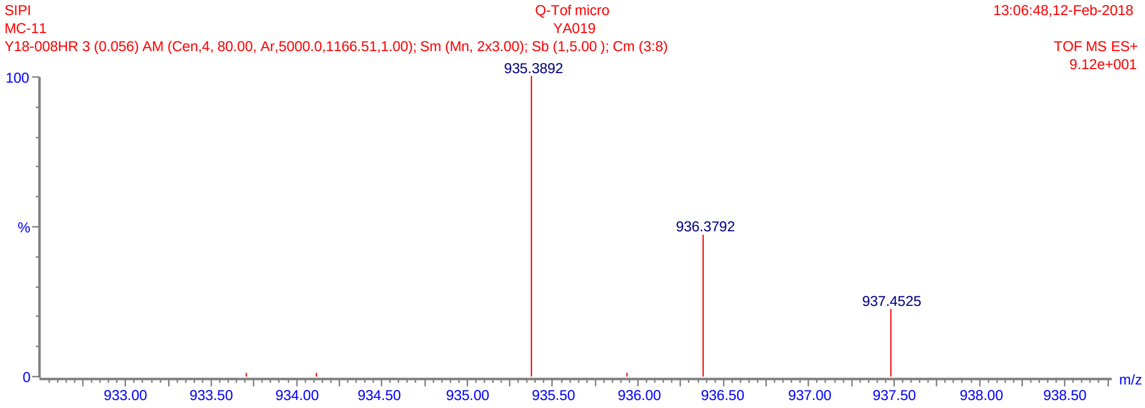

Figure S46. Positive ESI-MS spectrum of Compound **5**

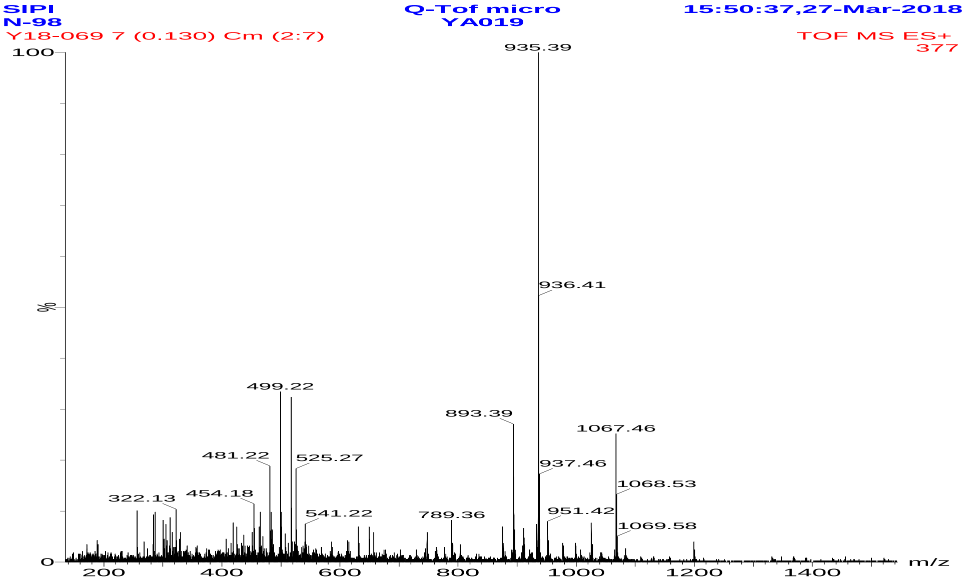

Figure S47. ^{1}H-NMR spectrum of Compound **5**

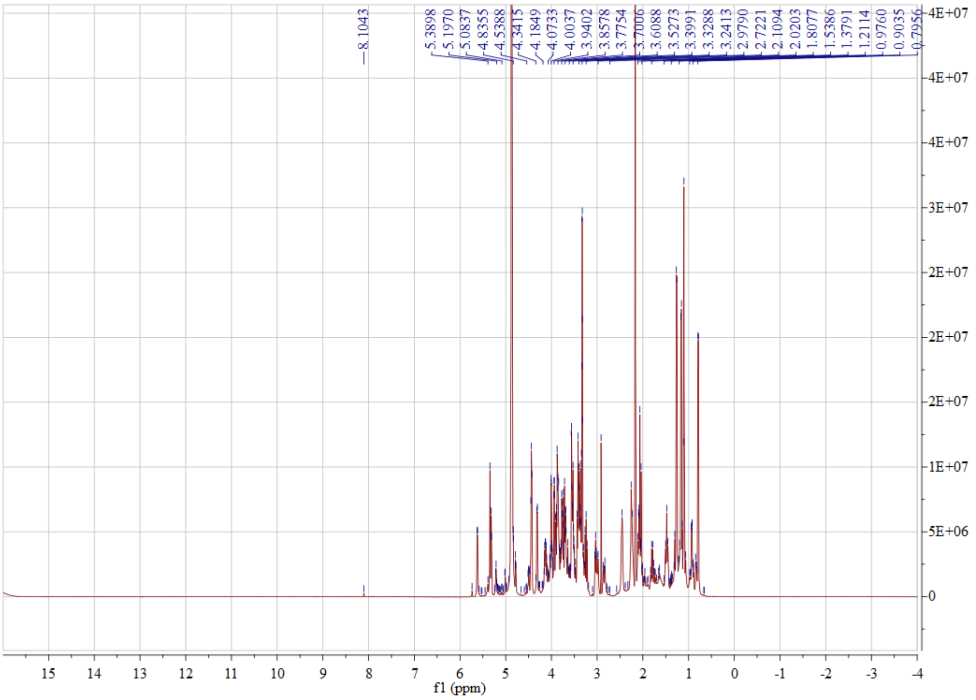

Figure S48. ^{13}C-NMR spectrum of Compound **5**

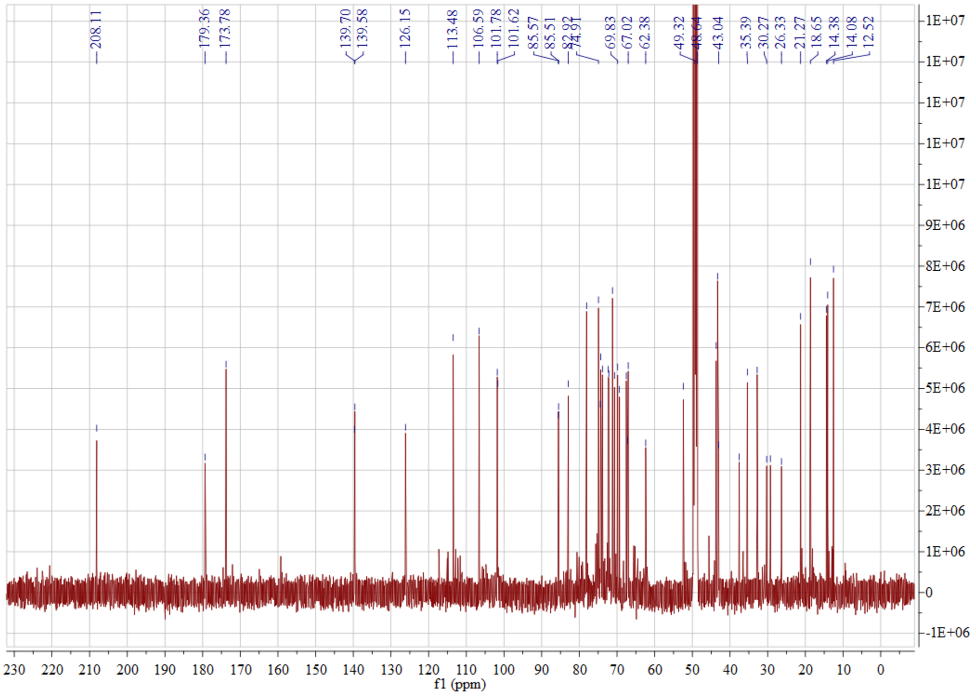

Figure S49. DEPT 90 spectrum of Compound **5**

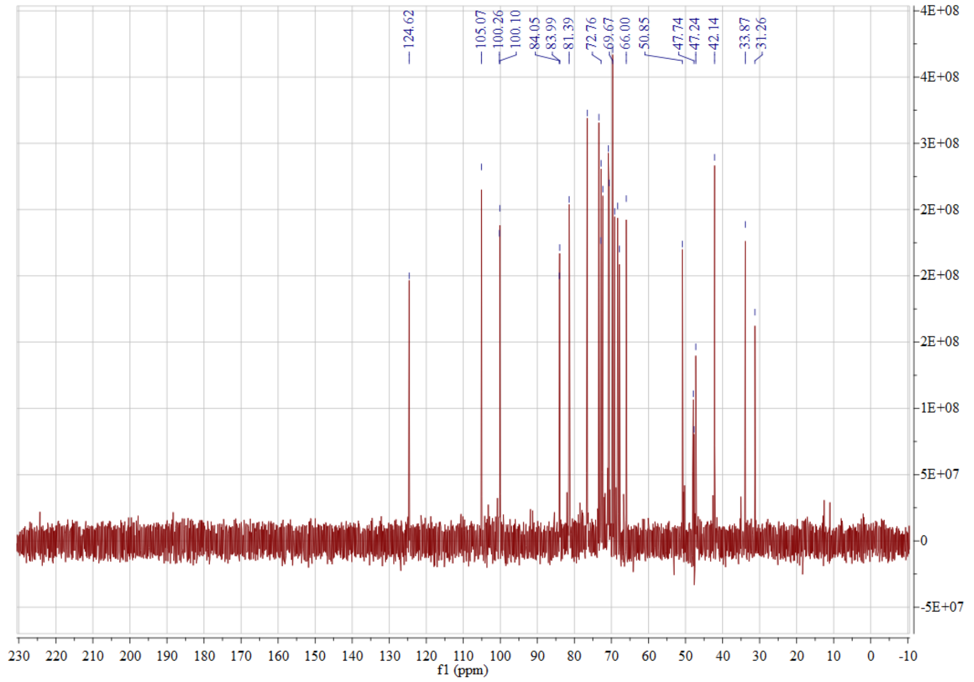

Figure S50. DEPT 135 spectrum of Compound **5**

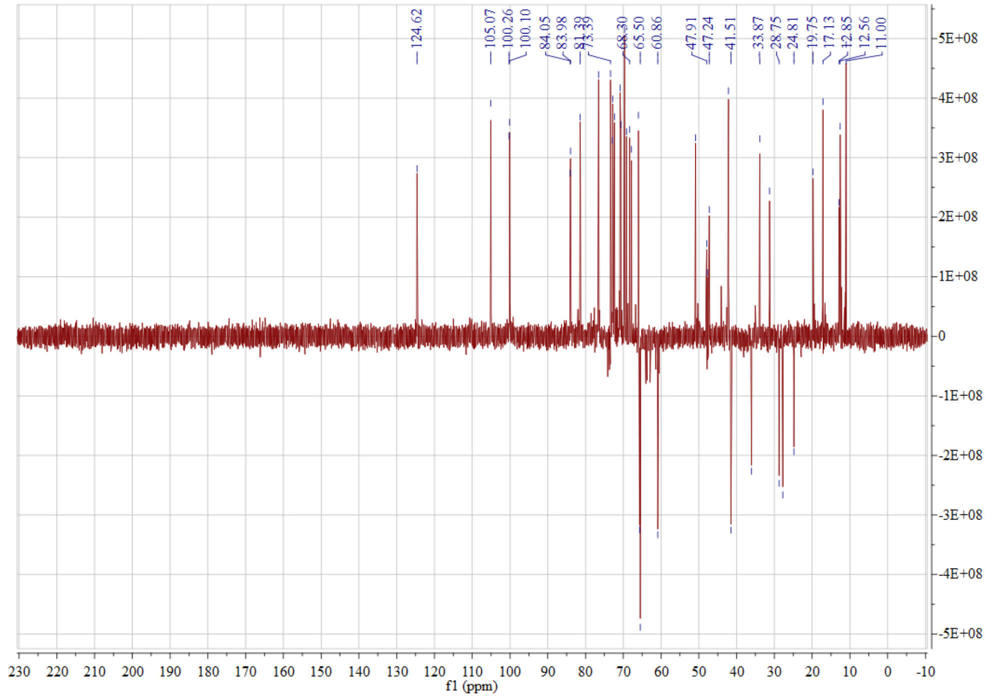

Figure S51. HSQC spectrum of Compound **5**

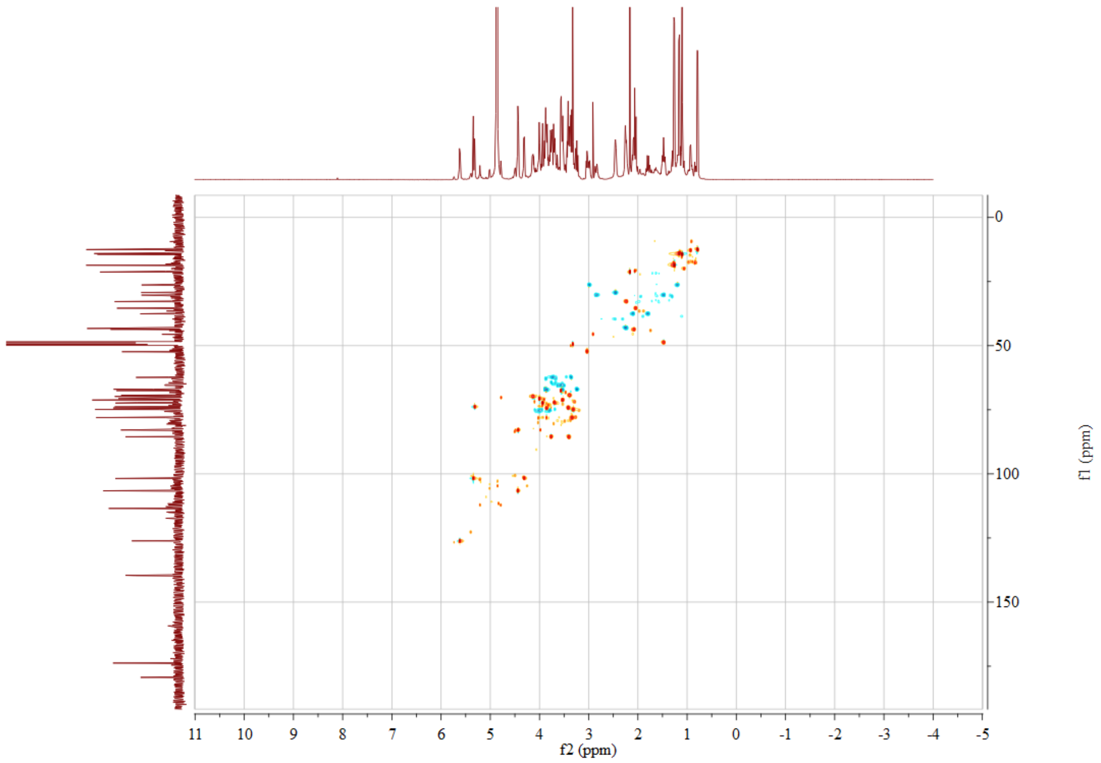

Figure S52. HMBC spectrum of Compound **5**

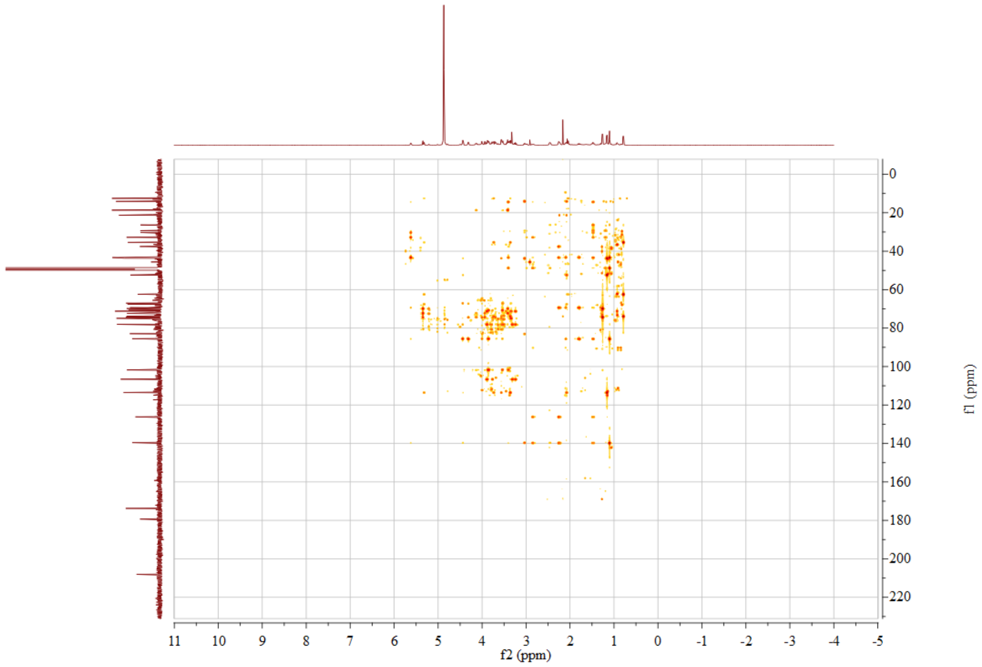

Figure S53. ^{1}H-^{1}H COSY spectrum of Compound **5**

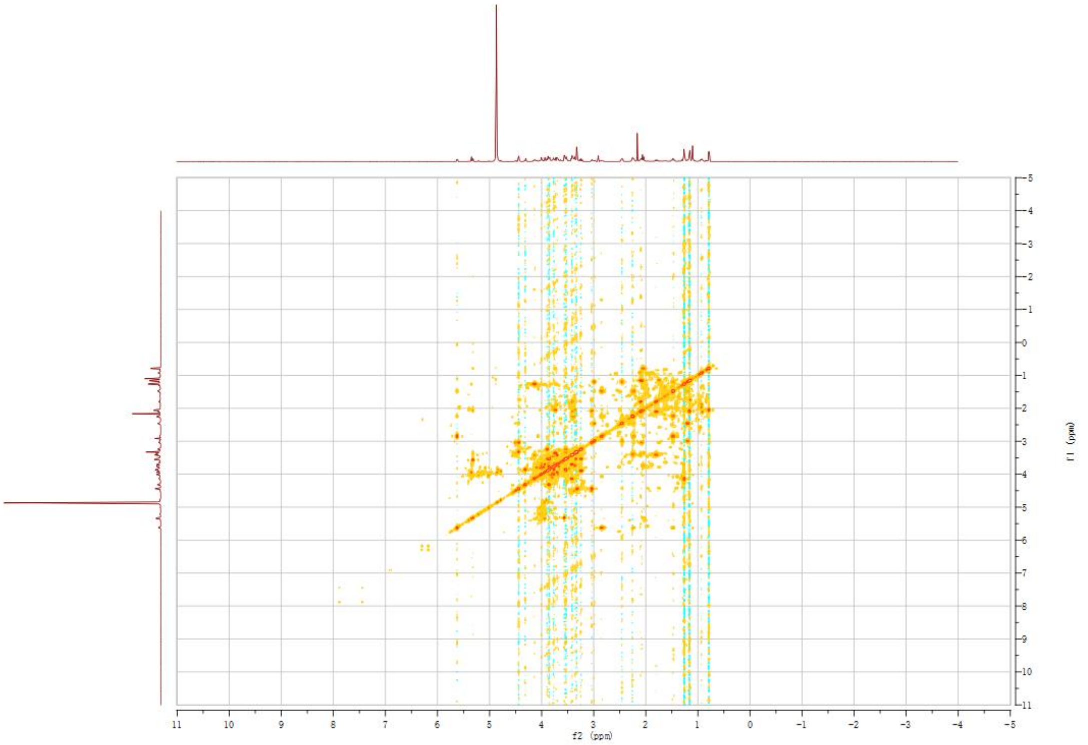

Figure S54. NOESY spectrum of Compound **5**

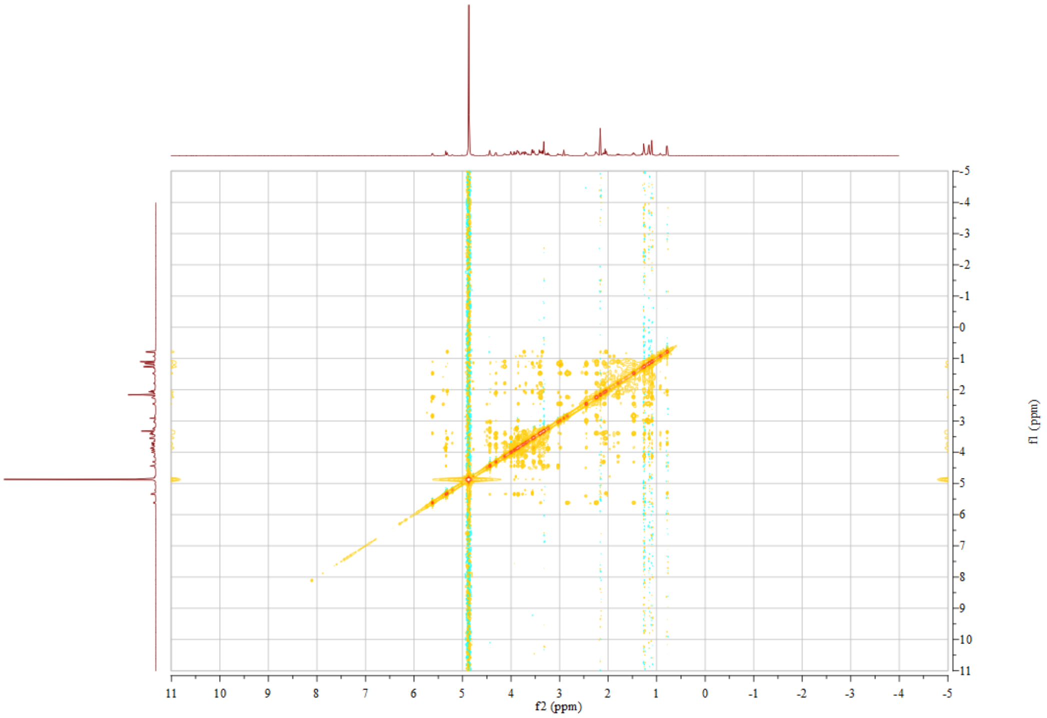

Figure S55. TOCSY spectrum of Compound **5**

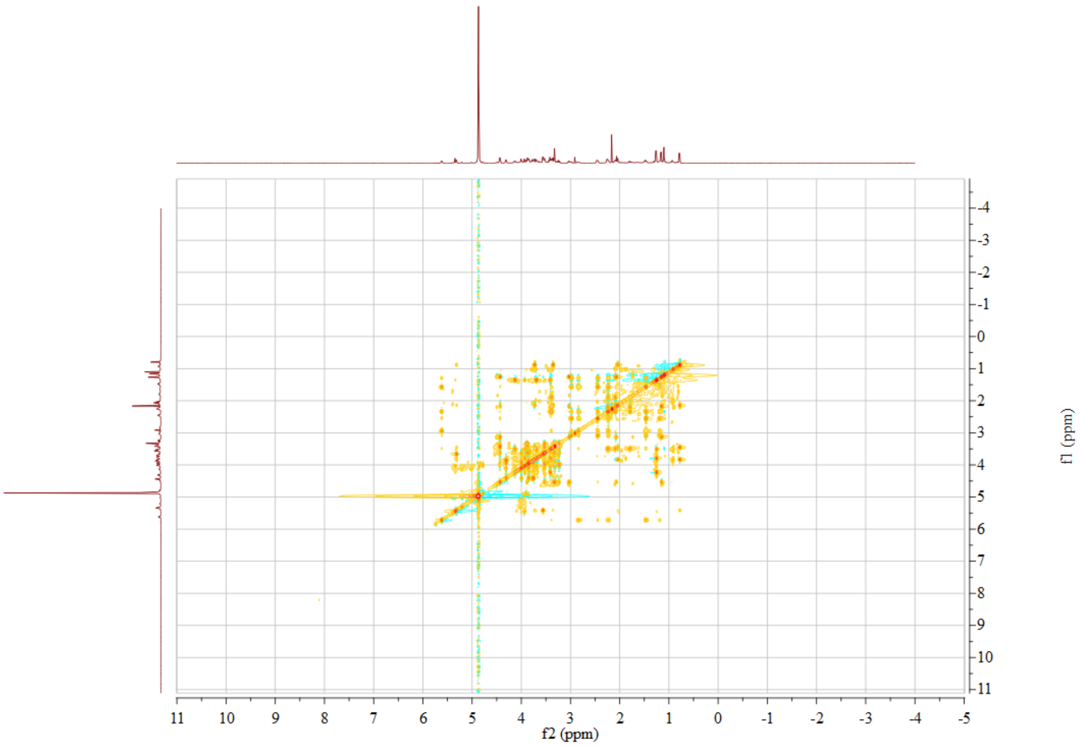

Supplement: Supplementary file 1 [file molecules-26-06366-s001.zip › molecules-1411270-supplementary.pdf]
